# Better Bioactivity, Cerebral Metabolism and Pharmacokinetics of Natural Medicine and Its Advanced Version

**DOI:** 10.3389/fphar.2022.937075

**Published:** 2022-06-27

**Authors:** Jiaxi Xie, Cailing Zhong, Tingting Wang, Dan He, Luyang Lu, Jie Yang, Ziyi Yuan, Jingqing Zhang

**Affiliations:** ^1^ Chongqing Research Center for Pharmaceutical Engineering, College of Pharmacy, Chongqing Medical University, Chongqing, China; ^2^ Biochemistry and Molecular Biology Laboratory, Experimental Teaching and Management Center, Chongqing Medical University, Chongqing, China; ^3^ College of Pharmacy, Southwest Minzu University, Chengdu, China

**Keywords:** cerebral metabolism, natural medicines, pharmacokinetics, pharmacodynamics, delivery systems

## Abstract

Currently, many people are afflicted by cerebral diseases that cause dysfunction in the brain and perturb normal daily life of people. Cerebral diseases are greatly affected by cerebral metabolism, including the anabolism and catabolism of neurotransmitters, hormones, neurotrophic molecules and other brain-specific chemicals. Natural medicines (NMs) have the advantages of low cost and low toxicity. NMs are potential treatments for cerebral diseases due to their ability to regulate cerebral metabolism. However, most NMs have low bioavailability due to their low solubility/permeability. The study is to summarize the better bioactivity, cerebral metabolism and pharmacokinetics of NMs and its advanced version. This study sums up research articles on the NMs to treat brain diseases. NMs affect cerebral metabolism and the related mechanisms are revealed. Nanotechnologies are applied to deliver NMs. Appropriate delivery systems (exosomes, nanoparticles, liposomes, lipid polymer hybrid nanoparticles, nanoemulsions, protein conjugation and nanosuspensions, etc.) provide better pharmacological and pharmacokinetic characteristics of NMs. The structure-based metabolic reactions and enzyme-modulated catalytic reactions related to advanced versions of NMs alter the pharmacological activities of NMs.

## Introduction

Cerebral diseases are usually caused by abnormal cerebral metabolism (anabolism and catabolism) of neurotransmitters, hormones, neurotrophic molecules, and other brain-specific chemicals (Graf et al., 2013). Alzheimer’s disease (AD), depression, cerebral injury and brain tumors are four major brain pathologies that induced by aberrant cerebral metabolism. AD is mainly caused by neuroinflammation (Angeloni et al., 2019), loss of neurons, and the accumulation of phosphorylated tau protein and amyloid plaques (Aβ) in the brain (Karch and Goate, 2015). AD has affected over 50 million people worldwide (Najm et al., 2020), leading to the progressive and irreversible loss of memory and other cognitive functions in patients (Nho et al., 2020). Depression results from dysregulated release of neurotransmitters. Depression has been found to occur in 14.8% of males and 14.1% of females worldwide (Kyu et al., 2018). It has become the third leading cause of disability (Corriger and Pickering, 2019). Traumatic brain injury (TBI) remains a common cause of disability and death worldwide (VanItallie, 2019) and leads to increased neuroinflammation (Karve et al., 2016). TBI is always accompanied by secondary injuries such as spastic cerebral palsy (Enslin et al., 2020), attention deficit hyperactivity disorder (Narad et al., 2018) and cerebral ischemia (Kaur and Sharma, 2018). Glioma, a malignant glial tumor, is the most common tumor in the central nervous system. Glioma has a higher rate of mortality than other tumors (Anjum et al., 2017) and is the second leading cause of death among central nervous system diseases (Bilmin et al., 2019). Targeting uncontrolled tumor proliferation in the brain (Shah and Kochar, 2018) by inhibiting tumor growth or engendering tumor apoptosis would be the most potent gliomas treatment.

Natural medicines (NMs) are known for their high availability, clear efficacy, and low toxicity and economic cost (Yang et al., 2020). Some NMs have been proven to have positive effects by regulating cerebral metabolism to ameliorate brain diseases. However, most NMs that affect cerebral metabolism (NMCs) have low solubility, low permeability and poor pharmacokinetic characteristics. Hence, loading NMCs with advanced drug delivery systems such as exosomes, nanoparticles and liposomes, provides ways to solve this problem.

Here, the relationships between NMCs found through available databases and cerebral metabolism are investigated. The solubility, permeability, molecular structure and molecular weight characteristics of various NMCs are presented. Drug delivery systems that enhance the pharmacokinetic and pharmacodynamic characteristics features of NMCs are reviewed. The structure-based *in-vivo* metabolic reactions modulated by metabolic enzymes and metabolites of NMCs are summarized.

## Effects of NMCS on Cerebral Metabolism

NMCs have effects on neurotransmitters. Neurotransmitters are chemicals released by axons to transfer information between neurons. Because of the substantial and unique roles neurotransmitters play in brain function, targeting neurotransmitter metabolism is considered a potent approach to treat neurological and psychiatric disorders (Hyman, 2005). Artemisinin, cannabidiol, geniposide and ginsenoside Rb1 are neuroprotective agents (Supplementary Table S1) (Liu W. et al., 2015; Watt and Karl, 2017; Zhao J. et al., 2018; Qiang et al., 2018). They treat AD and traumatic cerebral injuries and attenuate secondary injuries by inhibiting nitric oxide (NO) release. NO is a gas neurotransmitter. NO regulates the release of proinflammatory molecules, interacts with reactive oxygen species (ROS), promotes the formation of reactive nitrogen species (RNS), and ultimately causes cellular death (a hallmark of many neurodegenerative diseases and cerebral injuries) (Asiimwe et al., 2016). Evodiamine, icariin, curcumin and ferulic acid mitigate depressive symptoms by raising the levels of serotonin, norepinephrine, monoamine or dopamine (He et al., 2018; Jin et al., 2019; Sasaki et al., 2019; Fusar-Poli et al., 2020; Xie et al., 2020; Zhao et al., 2020; Xu et al., 2021). Baicalin controls symptoms of attention deficit hyperactivity disorder (ADHD) by increasing dopamine levels. Dysfunction of catecholamine and particular dopamine neuronal systems is considered a cause of ADHD (Zhou et al., 2019).

NMCs have effects on hormones. NMCs such as ferulic acid and icariin effectively relieve depressive-like behavior by decreasing the concentrations of corticosterone, adrenocorticotropic hormone (ACTH) and cortisol, which are also called the primary stress hormones. These NMs treat stress-induced depression caused by impaired regulation of the hypothalamic-pituitary-adrenal (HPA) axis (Jin et al., 2019; Zheng et al., 2019).

NMCs have effects on neurotrophic molecules. Neurotrophic molecules (also called neurotrophic factors) are molecules (mostly proteins) derived from neurons that facilitate the survival/differentiation of neurons (Unsicker, 2013). They are composed of neurotrophins, neuroregulatory cytokines, the fibroblast growth factor (FGF) family, the transforming growth factor-β (TGF-β) family, the insulin-like growth factor (IGF) family and other growth factors, such as vascular endothelial growth factor (VEGF). The metabolism of neurotrophic molecules affects the brain metabolism and thus affects neurodegenerative diseases and cerebral injuries.

NMCs have effects on neurotrophins. Neurtrophins are proteins that regulate the survival, growth and programmed cell death of neurons (Unsicker, 2013). Nerve growth factor (NGF) and brain-derived neurotrophic factor (BDNF) are two neurtrophins that play critical physiological roles in peripheral/central nervous system function. NGF and BDNF signaling also regulate neuropathic pain through receptors such as tropomyosin receptor kinase A (TrkA) and B (TrkB) (Khan and Smith, 2015). NMCs such as baicalin, curcumin and ferulic acid augment the levels of BDNF or the receptor TrkB to assuage depressive-like behaviors (Liu et al., 2017; Lu et al., 2019; Sasaki et al., 2019; Fusar-Poli et al., 2020), since depressive patients in the clinic are always found to have reduced BDNF levels in the peripheral system (Liu et al., 2017). Rutin mitigates cerebral ischemia injury by activating estrogen receptor-mediated BDNF-TrkB/NGF-TrkA signaling (Liu et al., 2018), and chlorogenic acid mitigates ischemic injury by increasing the level of NGF in brain tissue (Miao et al., 2017). Oleandrin has a neuroprotective effect in antitumor treatment by enhancing BDNF (Garofalo et al., 2017).

NMCs have effects on neuroregulatory cytokines. Neuroregulatory cytokines such as interleukin-6 (IL-6), ciliary neurotrophic factor (CNTF), leukemia inhibitory factor (LIF), cardiotrophin-1 and cardiotrophin-2 (CT-1 and CT-2), oncostatin-M and neuropoietin are useful in the treatment of neurodegenerative diseases and trauma (Unsicker, 2013). Anti-inflammatory cytokines prohibit the inflammation process, while pro-inflammatory cytokines promote the inflammation cascade (Boshtam et al., 2017). Artemisinin, cannabidiol, oxymatrine and geniposide treat AD by decreasing the expression of IL-6 (Liu et al., 2015b; Watt and Karl, 2017; Qiang et al., 2018; Chen Y. et al., 2019). Overexpression of proinflammatory cytokines provokes neurodegeneration induced by activated microglia, which are thought to clear the Aβ protein. Accordingly, accumulated Aβ leads to neuroinflammation, neuronal synapse loss and eventually AD (Kaur et al., 2019). The proinflammatory cytokines are released after middle cerebral artery occlusion (MCAO) catalyzed by focal cerebral ischemia/reperfusion (I/R) damages of blood brain barrier, cerebral edema and acute inflammation. Salvianolic acid B relieves cerebral injury by reducing IL-6 expression (Fan et al., 2018) Leonurine, baicalin, geniposide and ferulic acid (Jia et al., 2017; Zhao Y. et al., 2018; Guo et al., 2019; Zheng et al., 2019) reduce IL-6 expression to treat depression (Kim et al., 2016).

NMCs have effects on TGF-β and VEGF. TGF-β is involved in the development, differentiation, angiogenesis, apoptosis and survival of body cells. Anomalous expression or dysregulation of TGF-β leads to neurodegenerative disease, cancers, and so on (Cabello-Verrugio, 2018). TGF-β may act as a pro- or anti-inflammatory cytokine in different contexts. Salvianolic acid B increases TGF-β (acting as an anti-inflammatory factor) to palliate depressive-like behaviors in depressive patients (Zhang et al., 2016), whereas icariin reduces TGF-β (acting as a proinflammatory factor) to treat cerebral ischemia (Jin et al., 2019). VEGF is essential for vascular and nervous system development, and tanshinone IIA activates VEGF to prompt angiogenesis, axon growth, and neuronal survival and to protect nerve cells and resist apoptosis after brain damage (Zhang W. et al., 2017). Leonurine augments VEGF expression, which is conducive to the formation of nuclear factor erythroid 2-related factor 2 (Nrf-2), to treat cerebral ischemic stroke (Xie et al., 2019).

NMCs have effects on other brain-specific chemicals. These chemicals usually participate in regulating neurotransmitters, hormones, and neuroregulatory cytokines to modulate cerebral metabolism. In the treatment of AD, tanshinone IIA, ginsenoside Rd, cannabidiol, oxymatrine, cholic acid, vitamin A, puerarin, icariin, geniposide and curcumin prevent and ameliorate AD by diminishing Aβ deposition and tau protein phosphorylation (Liu et al., 2015a; Karch and Goate, 2015; Zeng J. et al., 2017; Tang and Taghibiglou, 2017; Watt and Karl, 2017; Yan et al., 2017; Yao et al., 2017; Chen Z. et al., 2019; Jin et al., 2019; Majid et al., 2019; He et al., 2020; Fu et al., 2021). Cannabidiol disrupts the Wnt/β-catenin pathway to inhibit tau protein phosphorylation (Watt and Karl, 2017). Ginsenoside Rd, salvianolic acid B and icariin can also treat AD by increasing the expression of α-secretase and soluble amyloid precursor protein alpha (sAPPα), which are negatively related to Aβ formation, or by decreasing the expression of β-secretase, γ-secretase, BACE1, sAPPβ, and amyloid precursor protein (APP), which stimulates Aβ production (Tang et al., 2016; Yan et al., 2017; Jin et al., 2019). Rhynchophylline treats AD by inhibiting erythropoietin-producing hepatocellular A4 (EphA4), which is key in synaptic loss and dysfunction and mediates Aβ (Fu et al., 2021). Capsaicin and salvianolic acid B inhibit AD by inhibiting glycogen synthase kinase 3 beta (GSK-3β), leading to a decrease in inflammatory signaling molecules and preventing tau hyperphosphorylation (Tang et al., 2016; Xu et al., 2017). Neuronal apoptosis plays crucial roles in AD treatment. Tanshinone IIA and ginsenoside Rb1 are AD medicines that upregulate the ratio of Bcl-2 (an antiapoptosis protein) to Bax (a proapoptotic protein) and downregulate caspase-3 (an effector of the main initiator in the apoptotic pathway) (Wang C. et al., 2018; He et al., 2020). Butylphthalide inhibits mitogen-activated protein kinases (MAPKs), which accelerate brain tissue apoptosis, to treat AD (Song et al., 2017). Tanshinone IIA prevents AD by preventing the abnormal expression of glucose regulated protein 78 (GRP78), eukaryotic initiation factor 2-alpha (eIF2α), inositol-requiring enzyme 1α (IRE1α) and activating transcription factor 6 (ATF6) to prevent endoplasmic reticulum (ER) stress, which would induce apoptosis and eventually AD through the CCAAT/enhancer-binding protein homologous protein (CHOP) and c-Jun N-terminal kinase (JNK) pathways, which are also hindered by tanshinone IIA (He et al., 2020). Geniposide activates the GLP-1R/AKT signaling pathway, which exerts neuroprotective effects against AD and depression by preventing apoptosis and inflammatory processes and promoting neurite outgrowth (Liu W. et al., 2015; Zhao J. et al., 2018). Moreover, as mentioned before, the proinflammatory process accelerates AD and the associated deterioration. Artemisinin, tetrandrine, cannabidiol, oxymatrine, and geniposide directly reduce the expression of proinflammatory cytokines such as IL-6, IL-1β, IL-17A, and TNF-α (Liu et al., 2015b; Watt and Karl, 2017; Qiang et al., 2018; Chen Y. et al., 2019; Ren et al., 2021) to control AD. Nuclear factor kappa-light-chain-enhancer of activated B cells (NF-κB), which is suppressed by artemisinin, tetrandrine, cannabidiol, and scutellarein (Watt and Karl, 2017; Qiang et al., 2018; Huang et al., 2019; Ren et al., 2021), and cyclooxygenase, which is impeded by geniposide (Liu et al., 2015b), are always involved in the control of proinflammatory cytokines and inflammatory responses to promote AD. Artemisinin reduces the expression of MyD88, a transducer in the proinflammatory pathway, to alleviate AD (Qiang et al., 2018). Artemisinin and geniposide suppress Toll-like receptor 4 (TLR4) to inhibit the NF-κB and MAPK signaling pathways to ameliorate AD (Liu W. et al., 2015; Qiang et al., 2018). Capsaicin restores the PI3K/AKT signaling pathway to treat T2D-induced AD, since damage to brain insulin signaling might cause AD (Xu et al., 2017). Cannabidiol inhibits S100 calcium-binding protein B (S100B), inducible nitric oxide synthase (iNOS) and glial fibrillary acidic protein (GFAP) to reduce reactive gliosis induced by Aβ (Watt and Karl, 2017).

Leonurine, salvianolic acid B, baicalin, geniposide, and ferulic acid (Zhang et al., 2016; Jia et al., 2017; Zhao Y. et al., 2018; Guo et al., 2019; Zheng et al., 2019) decrease the proinflammatory cytokines IL-1β and TNF-α or increase the antiinflammatory factors IL-10 and TGF-β to assuage depressive-like behavior. Leonurine and curcumin inhibit NF-κB (Jia et al., 2017; Fusar-Poli et al., 2020). Baicalin reduces TLR4 while augmenting the PI3K/AKT/FoxO1 pathway to mitigate depression (Guo et al., 2019). Icariin and curcumin decrease the level of corticotropin-releasing factor (CRF), a protein that leads to the release of cortisol and monoamine oxidase A and B and catalyzes the metabolism of norepinephrine, serotonin, and dopamine (Gu et al., 2017; Jin et al., 2019; Fusar-Poli et al., 2020). Icariin also restores the glucocorticoid receptor (GR) and serotonin 1A receptor levels, facilitating antidepressive behavior by improving HPA axis function (Jin et al., 2019). Baicalin and ferulic acid increase the levels of synaptic proteins, including postsynaptic density protein 95 and synapsin I, which are inactivated in depressive patients (Liu et al., 2017; Lu et al., 2019).

In addition to leading to AD and depression, the proinflammatory process also contributes to TBI and secondary injuries. Ginsenoside Rb1, salvianolic acid B, and icariin directly decrease the levels of proinflammatory factors, such as IL-1β and TNF-α (Zhao J. et al., 2018; Fan et al., 2018; Jin et al., 2019). Tanshinone IIA suppresses p-NF-κB, p-p38MAPK and iNOS to mitigate SCP (Zhang X. et al., 2017). Ginsenoside Rb1 decreases iNOS, and evodiamine and icariin reduce NF-κB to treat cerebral injury (Zhao et al., 2014; Zhao J. et al., 2018; Jin et al., 2019). Icariin also inhibits the degradation of NF-κB light polypeptide gene enhancer in B-cells inhibitor alpha (IκB-α, an inhibitor of NF-κB), and increases peroxisome proliferator-activated receptor-alpha (PPARα) and peroxisome proliferator-activated receptor-gamma (PPARγ) to upregulate antiinflammatory cytokines and downregulate proinflammatory factors (Morotti et al., 2017; Jin et al., 2019). Leonurine upregulates Nrf-2, which improves oxidative stress in cerebral ischemic stroke and benefits brain tissues by increasing VEGF levels (Xie et al., 2019). Ginsenoside Rb1 inhibits high-mobility group box 1 (HMGB1), a proinflammatory mediator, to disrupt the inflammatory signals (Zhao Y. et al., 2018). Tanshinone IIA activats the PI3K/AKT/mTOR pathway to protect HT-22 cells from oxidative stress injury (Zhu et al., 2017). Ginsenoside Rb1 is neuroprotective against cerebral ischemia by activating the P-AKT/P-mTOR signaling pathway and inhibiting the P-PTEN protein, which is an inhibitor of the PI3K/AKT signaling pathway (Guo et al., 2018). Rutin boosts the levels of estrogen receptor alpha and beta (ERα and ERβ), which modulate the growth, survival and metabolism of cells by regulating downstream targets and activating the BDNF-TrkB and NGF-TrkA signaling pathways, to mitigate cerebral ischemia injury (Liu et al., 2018). Chlorogenic acid increases hypoxia-inducible factor alpha (HIF-1α), which is neuroprotective against cerebral ischemia reperfusion injury by regulating erythropoietin (EPO), VEGF, glucose transporter 1 (GLUT-1) and adrenomedullin (ADM) (Miao et al., 2017). Evodiamine upregulates pAkt and pGSK3β by activating the AKT/GSK signaling pathway to exert anti-inflammatory effects against cerebral ischemia (Zhao et al., 2014). Salvianolic acid B reduced GFAP, ionized calcium-binding adaptor molecule 1, and caspase-3 to suppress astrocyte activation, which diminishes brain cell apoptosis (Fan et al., 2018).

Regarding glioma treatment, Δ9-tetrahydrocannabinol binds to G protein-coupled cannabinoid receptors 1 and 2 (CB1, CB2) to stimulate MAPK and endoplasmic reticulum stress-related pathways to reduce tumor growth (Scott et al., 2014). Salvianolic acid B stimulated intracellular ROS production and eventually caused apoptotic cell death in glioma U87 cells (Wang et al., 2013). Flavokawain B activated the ATF4-DDIT3-TRIB3-AKT-mTOR-RPS6KB1 signaling pathway in human glioblastoma multiforme cells to promote autophagy in glioma cells (Wang J. et al., 2018).

Although some of the mechanisms of effects of NCMs on these diseases remain unclear, and animal experiments are mainly performed for these NCMs, they have shown remarkable impact on the mitigation and prevention of AD, depression, TBI and its following injuries, and glioma.

## Solubility, Permeability and Structural Properties of NMCS

NMC absorption is largely contingent on solubility and permeability. Usually, higher solubility and permeability results in better absorption (Zeng M. et al., 2017; Mo et al., 2018; Yang et al., 2020). The dose number (D_0_) and oil-in-water partition coefficient (Log *p*) numerically represent the solubility and permeability, respectively. The *D*_0_ and Log *p* of a drug determine its biopharmaceutical classification in the Biopharmaceuticals Classification System (BCS) (Yang et al., 2020).

Most NMCs belong to BCS II, III, or IV, which tend to include drugs with low solubility, permeability, or both (Supplementary Table 2) (Charalabidis et al., 2019). NMCs with relatively low solubility, such as some terpenes, alkaloids, acids and esters, vitamins, flavonoids and phenylpropanoids, belong to BCS II, while NMCs with low permeability, such as other alkaloids, flavonoids, glycosides and phenylpropanoids, belong to BCS III. In addition, flavonoids such as puerarin and glycosides such as icariin belong to BCS IV since they have low solubility/low permeability. Obviously, NMCs with limited absorption would lead to further consequences such as poor pharmacokinetic properties and metabolism. Suitable drug delivery systems for NMCs are required to fix this dilemma.

## Improved Pharmacokinetics and Bioactivity of NMC Delivery Systems

The blood-brain barrier (BBB) helps to establish and maintain the microenvironment of the central nervous system (CNS) (Tsou et al., 2017; Liebner et al., 2018). The BBB only allows essential nutrients and certain molecules, such as O_2_, CO_2_, glucose and ethanol to enter (Tsou et al., 2017; Battaglia et al., 2018; Sharma et al., 2019). In the treatment of CNS diseases, it is a major challenge to make enough drug to across the BBB and achieve an effective concentration in the brain. NMC drug delivery systems (NMC-DDSs) have been developed to facilitate drug transport across the BBB and accumulation in the brain and to improve their efficacy in the CNS (Tables 1, 2) (Auffinger et al., 2013; Battaglia et al., 2018). The main NMC-DDSs include exosomes, nanoparticles, liposomes, lipid polymer hybrid nanoparticles (LPHNPs), nanoemulsions, protein conjugation and nanosuspensions.

**TABLE 1:** Characteristics of NMCs-DDS.

Drug	DDS	Administration route	Advantage	Main excipient	Preparation method	Characterization				References
						Particle size (nm)	Zeta potential (mV)	EE (%)	DL (%)	
Artemisinin	Nanostructured lipid carrier	—	Increase water solubility, site specificity, selective targeting, efficient penetration, glioma cell distribution and internalization, and effective delivery	Transferrin	Solvent evaporation method	145 ± 12.5	24.3 ± 1.5	82.3 ± 7.3	—	Emami et al. (2018)
Tanshinone IIA	Nanoparticle	i.v.	Prolong circulation time, increase plasma concentration, and have better brain delivery efficacy	Cationic albumin	Double emulsion/solvent evaporation method	122 ± 16	−17.8 ± 1.6	85.6 ± 3.2	5.86 ± 0.8	Liu et al. (2013)
	Nanoemulsion	i.v.	Prolong *in vitro* and vivo circulation time, and enhance the bioavailability	Tetramethylpyrazine	Shear stirring method	32.5	−2.78	95.26	—	Chen Y et al. (2019)
	Nanoparticle	i.v.	Better delivery efficacy	Cationic bovine serum albumin	Emulsification and solvent evaporation method	118 ± 14	−19.6 ± 1.4	83.2 ± 2.6	5.69 ± 0.6	Liu et al. (2013)
Capsaicin	Nanoparticle	i.v.	Be able to cross the blood-brain barrier and inhibit the growth of U251 cells	mPEG-PCL	Solvent diffusion method	121.3 ± 2.5	−9.1 ± 2.8	96 ± 5.1	9.4 ± 2.3	Jiang et al. (2015)
Salvianolic acid B	Nanoparticle	Brain injection	Sustain and prolong the *in vitro* release	Poly (ethyl-cyanoacrylate) coated with Tween 80	Emulsion polymerization method	288 ± 1.00	−8.38 ± 3.87	—	—	Grossi et al. (2017)
	Nanoparticle	Brain injection	Sustain and prolong the *in vitro* release	Poly (ethyl-cyanoacrylate)	Emulsion polymerization method	205 ± 2.00	−7.18 ± 2.84	98.70 ± 0.45	53.3 ± 0.24	Grossi et al. (2017)
Rutin	Lipid polymer hybrid nanoparticle	i.v.	Higher rutin bioavailability	Tween 80 coated PEG	Single-step nanoprecipitation technique	272.50 ± 3.39	−5.03 ± 0.18	64.32 ± 1.11	—	Ishak et al. (2017)
	Lipid polymer hybrid nanoparticle	i.v.	Higher rutin bioavailability	TPGS coated PEG	Single-step nanoprecipitation technique	203.00 ± 2.20	−2.52 ± 0.52	74.23 ± 2.14	—	Ishak et al. (2017)
	Lipid polymer hybrid nanoparticle	i.v.	Higher rutin bioavailability	Solutol HS 15 coated PEG	Single-step nanoprecipitation technique	232.4 ± 4.01	−1.76 ± 0.33	68.06 ± 1.50	—	Ishak et al. (2017)
	Nanoparticle	i.v.	Higher bioavailability; enhanced neurobehavioral activity, histopathology and reduced infarction volume effects	Chitosan	Ionic gelation method	92.28 ± 2.96	31.04 ± 1.91	84.98 ± 4.18	39.48 ± 3.16	Ahmad et al. (2016a)
Baicalin	Liposome	i.v.	Prolong the retention time *in vivo*, and increase the drug-concentration in the brain	—	Reverse evaporation method	160–190	−5.7	42 ± 1	—	Li et al. (2018)
	Cationic solid lipid nanoparticle	i.v.	Improve uptake of Baicalin	OX26 antibody	Emulsion evaporation–solidification at low temperature method	47.68 ± 1.65	−0.533 ± 0.115	83.03 ± 0.01	2.90 ± 0.01	Liu et al. (2015b)
Curcumin	Nanosuspension	i.v.	Improve the biodistribution of curcumin in the brain	TPGS	Probe sonicator technique	199 ± 2.5	−15.2 ± 3.3	—	—	Dibaei et al. (2019)
	Nanosuspension	i.v.	Improve the biodistribution of curcumin in the brain	Tween 80	High-pressure homogenizer technique	193 ± 8	−12.9 ± 1.7	—	—	Dibaei et al. (2019)
	Nanoparticle	i.n.	Enhance bioavailability	PNIPAM	Free radical polymerization	92.46 ± 2.8	−16.2 ± 1.42	84.63 ± 4.2	39.31 ± 3.7	Ahmad et al. (2016b)
	Exosome	i.v.	Enhance solubility, bioavailability, and stability and increase drug penetration across the BBB	—	—	117.4 ± 10.5	−4.9	84.8	15.1	Wang H et al. (2019)
	Exosome	i.v.	Improve safety and efficiency	c (RGDyK) peptide	—	145	−26.1	—	—	Tian et al. (2018)
	Exosome	i.v.	Increase drug penetration across the BBB	Superparamagnetic iron oxide	—	122.7 ± 6.5	−24.1 ± 2.2	—	—	Jia et al. (2018)
Rhynchophylline	Nanoparticle	i.v.	Better solubility and bioavailability and prolong circulation time	mPEG-PLGA	Nanoprecipitation method	145.2	—	60	10.3	Xu et al. (2020)

Abbreviations: c(RGDyK) peptide, cyclo(Arg-Gly-Asp-D-Tyr-Lys) peptide; DL, drug loading; EE, encapsulation efficiency; i.n., intranasal injection; i.p., intraperitoneal injection; i.v., intravenous injection; mPEG-PCL, methoxy polyethylene glycol-poly(caprolactone); PNIPAM, ploly-N-isopropylacrylamide; Solutol HS 15, polyethylene glycol-15-hydroxy stearate; TPGS, D-a-Tocopherol polyethylene glycol 1000 succinate; Tween 80, polyethylene glycol sorbitan monooleate.

Note: — refers to not reported.

**TABLE 2:** | Pharmacokinetic characteristics of NMC-DDS.

NMCs	Formulation	Administration route	Dosage (mg/kg)	Animal (number)	Pharmacokinetics parameters								References
					*AUC*_*0-t*_ (μg·h·ml^−1^)	*AUC*_*0-*∞_ (μg·h·ml^−1^)	*C*_max_ (μg·ml^−1^)	*T*_max_ (h)	*t*_1/2_ (h)	*MRT*_*0-t*_ (h)	*MRT*_*0-*∞_ (h)	*Cl* (L/h·kg)	
Tanshinone IIA	Nanoparticle	i.v.	10	Rats (6)	—	4.83 ± 0.49	—	0.54	8.29 ± 1.37	—	7.96 ± 0.68	0.31 ± 0.06	Liu et al. (2013)
	Nanoemulsion	i.v.	5	Rats (6)	4.55 (0–6 h)	8.03	3.52 ± 0.75	—	5.77	1.96 (0–6 h)	7.35	—	Chen Y et al. (2019)
	Nanoparticle	i.v.	10	Rats (10)	—	4.71 ± 0.58	—	—	8.17 ± 1.28	—	7.89 ± 0.74	0.28 ± 0.05	Liu et al. (2013)
Capsaicin	Nanoparticle	i.v.	—	—	—	—	—	—	—	—	—	—	Jiang et al. (2015)
Salvianolic acid B	Nanoparticle	i.p.	—	—	—	—	—	—	—	—	—	—	Grossi et al. (2017)
	Nanoparticle	i.p.	—	—	—	—	—	—	—	—	—	—	Grossi et al. (2017)
Rutin	Tween 80-lipid polymer hybrid nanoparticle	i.v.	5	Rats (6)	1.14 ± 0.27^a^ (0–48 h)	1.59 ± 0.56^a^	0.57 ± 0.13^b^	0.25 ± 0.00	—	—	4.41 ± 1.18	—	Ishak et al. (2017)
	TPGS-lipid polymer hybrid nanoparticle	i.v.	5	Rats (6)	1.11 ± 0.31^a^ (0–48 h)	1.80 ± 0.41^a^	0.67 ± 0.34^b^	1.17 ± 0.42	—	—	6.26 ± 4.25	—	Ishak et al. (2017)
	Solutol HS 15-lipid polymer hybrid nanoparticle	i.v.	5	Rats (6)	1.31 ± 0.53^a^ (0–48 h)	1.50 ± 0.47^a^	0.66 ± 0.33^b^	1.17 ± 0.44	—	—	3.52 ± 0.78	—	Ishak et al. (2017)
	Nanoparticle	i.n.	10	Rats (6)	0.35 (0–24 h)	—	1.45	2.00	43.68 ± 11.63	—	—	—	Ahmad et al. (2016a)
	Nanoparticle	i.v.	10	Rats (6)	8.50 E-02 (0–24 h)	—	0.39	2.00	39.01 ± 7.41	—	—	—	Ahmad et al. (2016b)
Baicalin	Liposome	i.v.	18	Rats (5)	88.27 (0–8 h)	103.61	52.48 ± 8.18	—	3.17	2.33 (0–6 h)	3.84	2.91 ± 0.25^c^	Li et al. (2018)
	Cationic solid lipid nanoparticle	i.v.	4.42	Rats (3)	—	2.68E-02	2.32E-02	0.94 ± 0.43	—	—	—	—	Liu et al. (2015b)
Curcumin	TPGS-nanosuspension	i.v.	10	Rats (6)	0.89 (0–6 h)	0.96	1.12	0.50	1.45 ± 0.180	0.61 ± 0.050 (0–6 h)	—	0.011 ± 0.001^d^	Dibaei et al. (2019)
	Tween 80-nanosuspension	i.v.	10	Rats (6)	1.79 (0–6 h)	1.87	1.31	0.75	1.94 ± 0.292	0.76 ± 0.194 (0–6 h)	—	0.006 ± 0.001^d^	Dibaei et al. (2019)
	PNIPAM- Nanoparticle	i.n.	0.1	Rats (6)	2.43^e^ (0–24 h)	—	2.36 E-03	1.00	7.70	—	—	—	Ahmad et al. (2016a)
	Exosome	i.v.	0.4	Rats (3)	9.03 (0–24 h)	—	0.91	—	9.02	—	—	3.67 E-02	Wang X et al. (2019)
	cRGD-Exosome	i.v.	—	—	—	—	—	—	—	—	—	—	Tian et al. (2018)
	RGE-Exosome-SPION	i.v.	—	—	—	—	—	—	—	—	—	—	Jia et al. (2018)
Rhynchophylline	Tween 80- Nanoparticle	i.v.	1	Rats (6)	—	0.41	0.67	—	1.48	—	—	1.94	Xu et al. (2020)

Abbreviations: cRGD, cyclo(Arg-Gly-Asp-D-Tyr-Lys)-conjugated; i.n., intranasal injection; i.p., intraperitoneal injection; i.v., intravenous injection; PNIPAM, ploly-N-isopropylacrylamide; RGE, neuropilin-1-targeted peptide; Solutol HS 15, polyethylene glycol-15-hydroxy stearate; SPION, superparamagnetic iron oxide nanoparticles; TPGS, D-a-Tocopherol polyethylene glycol 1000 succinate; Tween 80, polyethylene glycol sorbitan monooleate.

Note: — refers to data not reported.

a mg·g^−1^·h.

b mg·g^−1^.

c ml/(min·kg).

d (mg/kg)/(ng/ml)/h.

e (ng·min/ml).

Exosomes are cell-derived nanovesicles (Kojima et al., 2018), currently considered to be specific secretory vesicles for intercellular communication (Milane et al., 2015). Exosomes can disrupt the intact BBB by transcytosis (Morad et al., 2019), easily penetrate the BBB and safely delivers therapeutic drugs (Zhu et al., 2019). In addition, exosomes might have targeting capabilities after cell source selection and membrane modification (Zhu et al., 2019). Curcumin-primed exosomes secreted by mouse macrophage cells were fabricated to prevent neuronal death and alleviate AD symptoms (Wang H. et al., 2019). Curcumin-primed exosomes led to curcumin acumination 6.5 times higher than that of free curcumin in the brain, 2.5 times higher in the liver and 2.0 times higher in the lung (Wang X et al., 2019). Curcumin and superparamagnetic iron oxide nanoparticles were loaded into exosomes and conjugated with neuropilin-1-targeted peptide by click chemistry to obtain glioma-targeting exosomes with imaging and therapeutic functions (Jia et al., 2018). Compared with free exosomes, target ligand-modified exosomes markedly improved the brain targeting and circulation time of curcumin in the body (Jia et al., 2018). A functional ligand, (cyclo (Arg-Gly-Asp-D-Tyr-Lys) peptide, was conjugated with the bioorthogonal copper-free azide alkyne cyclo-addition (click chemistry) method to form mesenchymal stromal cell-derived exosomes to deliver curcumin to the brain (Tian et al., 2018).

The mechanism by which nanoparticles penetrate the BBB is still not very clear. Currently, the relative theories are listed as follows (Morad et al., 2019; Akel et al., 2021; Alotaibi et al., 2021; Han and Jiang, 2021; Hou et al., 2022): 1) The phagocytosis of nanoparticles by cerebral vascular endothelial cells allows the drug to be released and diffused into the brain; 2) The adsorption of capillary walls prolongs the residence time of drugs in the brain, thereby increases the amount of drugs entering the brain; 3) Nanoparticles open the tight junctions of capillary epithelial cells, and drugs penetrate into the brain from the open gaps; 4) The effect of some modifications of nanoparticles such as polysorbate 80, can efficiently inhibit the efflux pump p-gp glycoprotein. Rutin-encapsulated chitosan nanoparticles were fabricated via an ionic gelation method. After nasal administration, the *C*_max_, *t*_*1*/*2*_ and *AUC* in the brain of these nanoparticles were 6-, 1- and 7.3-fold higher than those of free rutin, respectively, the drug targeting efficiency increased by 2.3-fold, and the therapeutic effect increased accordingly (Ahmad et al., 2016b). Poly-N-isopropylacrylamide nanoparticles containing curcumin, demethoxycurcumin and bisdemethoxycurcumin were prepared by free radical polymerization. These nanoparticles increased the *C*_max_, *t*_1/2_ and *AUC* of the three drugs in the brain by approximately 4-, 9- and 5-fold, respectively (Ahmad et al., 2016a). A biodegradable methoxy polyethylene glycol-poly (caprolactone) amphiphilic block copolymer was used to prepare nanoparticle-loaded capsaicin for targeted treatment of glioma. These nanoparticle s had satisfactory slow-release features (Jiang et al., 2015). Rhynchophylline-loaded methoxy poly (ethylene glycol)-poly (DL-lactide-co-glycolic acid) nanoparticles coupled with Tween 80 were used for brain-targeted delivery (Xu et al., 2020).

Liposomes are nontoxic and have good biocompatibility and biodegradability (Pattni et al., 2015). Their phospholipid bilayer structure made them compatible with the lipid layer of the BBB and helped the drug enter the brain (Pattni et al., 2015; Agrawal et al., 2017). In addition, liposomes can be modified with different substances to achieve the ability to cross the BBB. By attaching lipid molecules to neurotransmitters, the resulting neurotransmitter lipidoids can be incorporated into drug-encapsulating liposomes, and give the liposomes ability to penetrate the BBB (Ma et al., 2020). There are various apolipoproteins in plasma that can cross the BBB, and one of the clearance mechanisms of Aβ protein in the brain is through the lipid binding of various apolipoproteins (such as ApoE, ApoA1 and ApoJ). When the receptor-binding region is exposed, it is mediated by the corresponding receptor on the BBB to the periphery. Because the related receptors can be transported in both directions, the peripheral ligands can also be transported to the brain, so that the drug can be transported to the brain to play a role (Zhang et al., 2019). Liposomes improved the lipophilicity of baicalin and further improved its pharmacokinetics in the brain. The *C*_max_ and *AUC* values of MCAO rats administered with baicalin-loaded liposomes were significantly greater than those of rats administered with baicalin; moreover, the *MRT* increased 2.14-fold, the *t*_1/2_ increased 2.87-fold, and the renal clearance rate decreased 8.08-fold. The pharmacokinetic parameter improvements led to prolonged retention time and enhanced therapeutic efficacy (Li et al., 2018).

LPHNPs are highly scalable, biodegradable nanocarriers composed of a layer of lipid-coated polymeric cores (polylactic-co-glycolic acid, polyglutamic acid, polylysine, PEG, etc.) (Dehaini et al., 2016; Ishak et al., 2017; Mukherjee et al., 2019). LPHNPs combined with liposomes and nanoparticles have advantages. Rutin delivered by LPHNPs coated with three surfactants, Tween 80, D-α-tocopheryl polyethylene glycol 1000 succinate (TPGS) and Soluted H55 had 160-, 98- and 159-fold higher bioavailability than free rutin, respectively (Ishak et al., 2017).

Nanoemulsions are nanosized droplets with high surface areas (Espinoza et al., 2019), so they have been used to solve drug solubility and stability problems (Bonferoni et al., 2017). The nanoemulsion mainly delivers drugs to the brain by adding excipients that increase BBB permeability or inhibit efflux proteins. The oil-in-water nanoemulsions for codelivery of tanshinone IIA and tetramethylpyrazine had the ability to pass through the BBB and target the brain. The *AUC*s of tanshinone IIA/tetramethylpyrazine or tanshinone IIA nanoemulsions were 6.98- and 5.83-fold higher than those of tanshinone IIA solution, respectively. The MRTs of two formers (117.68 and 123.29 min) were much longer than the latter (56.66 min). The *t*_1/2_ of the two nanoemulsions were 7.8- and 6.48-fold longer than that of the solution (Chen Z. et al., 2019). The resveratrol nanoemulsion was prepared by adding non-ionic surfactants Pluronic and Cremophor EL as emulsifier. The nanoemulsion was administered through nose to target the brain for AD treatment. The nanoemulsion increased the intracranial concentration of resveratrol by ∼87% and the AUC value by ∼92% (Kotta et al., 2021).

Proteins such as specific receptors (e.g., transferrin receptor) (Emami et al., 2018; Johnsen et al., 2018) and transporters expressed on the luminal side of brain endothelial cells help drugs cross the BBB through receptor-mediated endocytosis (Zuchero et al., 2016; Johnsen et al., 2018). Transferrin-coupled nanoliposomes were prepared to deliver artemisinin to the brain in a targeted manner (Emami et al., 2018). OX26 monoclonal antibody-conjugated cationic solid lipid nanoparticles were fabricated to improve baicalin distribution within the brain. The *AUC* and *C*_max_ values of baicalin nanoparticles were 11.08- and 7.88-fold higher than those of baicalin solution, respectively (Liu et al., 2015). Bovine serum albumin-conjugated cationic PEGylated nanoparticles containing tanshinone IIA had a 3.4-, 2.95- and 2.37-fold higher *AUC*, *t*_1/2_ and MRT than free tanshinone IIA, respectively (Liu et al., 2013).

Tween 80 and TPGS were separately used to coat on the surface of curcumin nanosuspensions by physical adsorption using a high-pressure homogenizer and a probe sonicator. The curcumin delivered by the nanosuspensions had almost 2-fold higher bioavailability than free curcumin (Dibaei et al., 2019).

## Metabolic Pathway and Metabolic Enzymes

It is vital to review the reactions and metabolites of NMCs (Supplementary Table 3). Most NMs are metabolized through chemical reactions by enzymes, which can cause them to become more active, less active, inactive, innocuous, or even noxious (Mo et al., 2018). Sometimes multiple metabolites are formed simultaneously, further experiments for the metabolism of NCMs are still needed.

In NMC metabolism, phase I reactions are involved in oxidation, reduction, and hydrolysis and are mediated by enzymes such as cytochrome P450 enzymes. Nonpolar functional groups on NMCs are changed into polar molecules (Iyanagi, 2007). Reactions in phase I include the followied chemical reaction (Eq. 1):

$$O_{2}+\;NADPH\;+\;H^{+}\;+\;RH\;\to \;NADP^{+}\;+\;H_{2}O\;+\;ROH$$ (1)

Oxidation reactions result in the addition of oxygen or the removal of hydrogen and encompass hydroxylation, dehydrogenation, and demethylation, among which hydroxylation is the most common reaction. Hydroxylation involves the addition of hydroxyl groups to aromatics, alkanes, or cycloalkanes. In the case of tanshinone IIA, two hydroxyl groups are directly added to the parent drug molecule, and the metabolite has been shown to be favorable in treating AD (Liang et al., 2019). Under catalysis by the metabolic enzyme CYP3A4, Δ9-tetrahydrocannabinol is oxidized to 8α (or β)-OH-Δ9-tetrahydrocannabinol, and further oxidized to 8-keto-Δ9-tetrahydrocannabinol (Dinis-Oliveira, 2016). The alkyl side chain of capsaicin is oxidized to a hydroxyl chain (Rollyson et al., 2014). Each phenyl group of evodiamine is oxidized to hydroxyl groups (Wang Y. et al., 2018). The metabolic enzymes CYP3A4 and CYP2E1 are the primary enzymes that hydroxylate butylphthalide (Diao et al., 2013; Diao et al., 2015). Additionally, dehydrogenation of hydroxyl groups to carbonyl groups or alkyl groups to alkenyl groups is found in NMC metabolism. The hydroxyl group on the cyclohexane of cholic acid is dehydrogenated to a carbonyl group by the enzyme CYP3A4 (Funabashi et al., 2020); the hydroxyl side chain of vitamin A is oxidized to a carbonyl group (Libien et al., 2017), and vitamin A was oxidized to all-trans-retinoic acid by retinal dehydrogenases (Clugston and Blaner, 2014). Leonurine is demethylated, which converts the methoxy group to a hydroxyl group (Zhu et al., 2014). Conversely, the addition of hydrogen or the removal of oxygen results in increased reduction reactions. Hydrogenation occurs to salvianolic acid B and ferulic acid when a double carbon is broken due to the addition of hydrogen (Wang et al., 2016; Zhang et al., 2022). Dehydroxylation entails the removal of hydroxyl groups, to further reduce the reduced metabolites of Salvianolic acid B (Zhang et al., 2022). The alkenyl group of ferulic acid is reduced to an alkyl group (Zhao et al., 2015); oxymatrine is reduced by CYP3A4 to matrine (Liu et al., 2015b). In hydrolysis, when reacting with water, compound bonds are broken to produce two compounds: one is bound with hydrogen cleaved from water molecules, and the other is bound with hydroxide. Ginsenoside Rb1 and Rd and puerarin are deglycosylated to lose one or two glucose molecules (Shang et al., 2017; Zhang et al., 2021); baicalin undergoes deglycosylation to form baicalein (Wang et al., 2017). Capsaicin is hydrolyzed to vanillylamine in the liver and skin (Rollyson et al., 2014); rutin undergoes hydrolysis to form metabolites such as quercetin and 3,4-dihydroxytoluene, 3,4-dihydroxyphenylacetic acid and 3,4-dihydroxybenzoic acid (Morales et al., 2018); and chlorogenic acid is hydrolyzed into caffeic acid and quinic acid by esterase (Choi et al., 2018). Geniposide is hydrolyzed to genipin through β-glucuronidase (Zhang W. et al., 2017).

In phase II reactions, NMCs undergo conjugation reactions, including glucuronidation, glycosylation, methylation, sulfonation, sulfation, cysteine conjugation, glucuronide conjugation and glucopyranoside conjugation, through metabolic enzymes such as UDP-glucuronosyltransferases (UGTs), sulfotransferases (SULTs), glutathione S-transferases (GSTs) (Iyanagi, 2007). Tanshinone IIA, geniposide, Δ9-tetrahydrocannabinol and puerarin undergo glucuronidation, which attaches a glucuronide (Dinis-Oliveira, 2016; Zhang X. et al., 2017; Shang et al., 2017; Liang et al., 2019). Salvianolic acid B, rutin, scutellarein, baicalin and puerarin are conjugated with methyl groups via methyltransferase (Shi et al., 2015; Gimenez-Bastida et al., 2017; Shang et al., 2017; Wang et al., 2017; Zhang et al., 2022). Leonurine, geniposide, and puerarin undergo sulfonation by SULTs (Zhu et al., 2014; Zhang W. et al., 2017; Shang et al., 2017); ferulic acid also needs SULTs to undergo sulfation (Wang et al., 2016). Conjugation of glucuronide to evodiamine (Wang C. et al., 2018) or glucopyranoside to baicalin (Wang et al., 2017) also occurs in phase II reactions.

## Conclusion and Future Prospect

**FIGURE 1:**
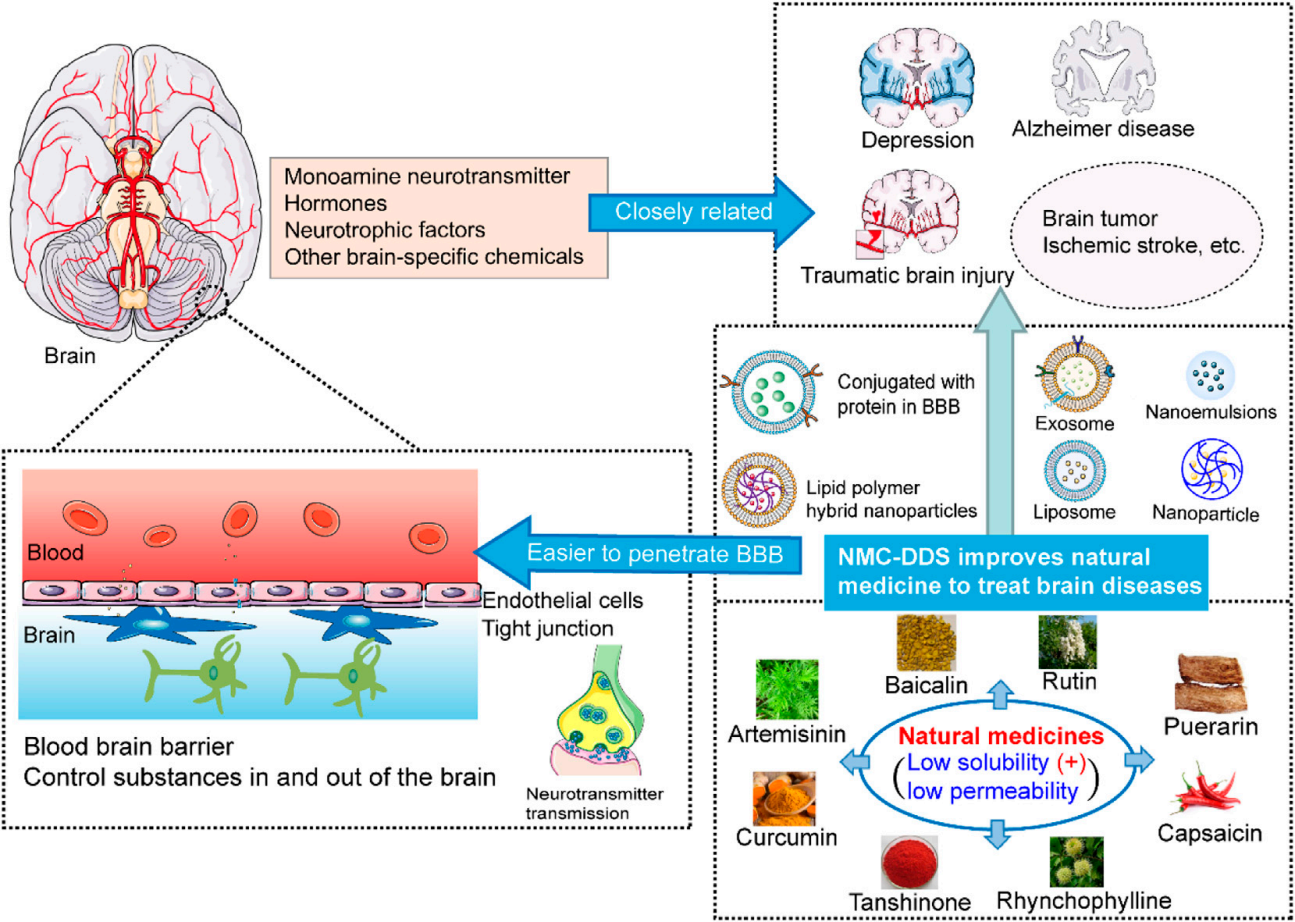
The schematic diagram for natural medicine delivery systems to improve bioactivity, increase metabolism and pharmacokinetic characteristics.

NMCs have appealing benefits as cerebral disease-treating drugs due to their effects on the metabolism of neurotransmitters, hormones, neurotrophic molecules, and other brain-specific chemicals in addition to their low cost, low toxicity, and obvious efficacy. Although the bioavailability/absorption of most NMCs is unsatisfactory, appropriate delivery systems such as novel nanosystems including exosomes, nanoparticles, LPHNPs, nanoemulsions, protein conjugation and nanosuspensions, provide better pharmacological and pharmacokinetic characteristics for NMCs. In addition, the structure-based metabolic reactions of NMCs, which produce more active, less active, inactive, innocuous, or even noxious metabolites, alter the pharmacological activities of NMCs (Figure 1). NMCs commonly undergo oxidation, reduction, hydrolysis and conjugation reactions, and metabolic enzymes such as cytochrome P450 enzymes, UGTs, and SULTs are needed in some cases. However, the metabolism and pharmacokinetics data for NMCs are still very limited.

In order to achieve clinical transformation of NMCs and overcome the key challenges ahead, the scientists may focus on the formulation prescription, industrial preparation, stability investigation and toxicity evaluation in the future. Since most of the current pharmacokinetic/pharmacological studies are based on animal experiments, more clinical evidence is needed for further application. NMCs are effective in cerebral-related disorders. They are strong candidates for clinical therapy of cerebral diseases. There have been some progress by now. GV-971 is a sodium oligomannate, which is derived from marine algae. GV-971 was first approved in China for marketing as a drug to mitigate AD. GV-971 (Syed, 2020) inhibits Aβ accumulation and decreases Aβ aggregates toxicity (Wang et al., 2020). Also, in support of the theory of the association between gut dysbiosis and AD, GV-971 ameliorates gut dysbiosis and suppresses neuroinflammation to improve cognition in AD (Wang H et al., 2019). Although debates exist about this drug, it is expected to have good prospects due to the safety and tolerance data obtained from phase III clinical trial evidence (Wang et al., 2020).

## Glossary

ACTH: Adrenocorticotropic hormone
Aβ: Amyloid plaques
AD: Alzheimer’s disease
ADM: Adrenomedullin
APP: Amyloid precursor protein
ATF6: Activating transcription factor 6
AUC: Area under the plasma concentration-time curve
BBB: Blood-brain barrier
BCS: Biopharmaceutics classification system
BDNF: Brain-derived neurotrophic factor
CB1: Cannabinoid receptor 1
CB2: Cannabinoid receptor 2
CHOP: CCAAT/enhancer-binding protein homologous protein
Cl: Clearance
C_max_: Maximum plasma
CNS: Central nervous system
CNTF: Ciliary neurotrophic factor
CRF: Corticotropin-releasing factor
CT-1: Cardiotrophin-1
CT-2: Cardiotrophin-2
D_0_: Dose number
eIF2α: Eukaryotic initiation factor 2-alpha
EphA4: Erythropoietin-producing hepatocellular A4
EPO: Erythropoietin
ER: Endoplasmic reticulum
ERα: Estrogen receptor alpha
ERβ: Estrogen receptor beta
FGF: Fibroblast growth factor
GFAP: Glial fibrillary acidic protein
GLUT-1: Glucose transporter 1
GR: Glucocorticoid receptor
GRP78: Glucose regulated protein 78GSK-3β
GSK-3β: Glycogen synthase kinase 3 beta
GSTs: Glutathione S-transferases
HIF-1α: Hypoxia-inducible factor alpha
HMGB1: High-mobility group box 1
HPA: Hypothalamic-pituitary-adrenal
IGF: Insulin-like growth factor
IκB-α: B-cells inhibitor alpha
IL-6: Interleukin-6
iNOS: Inducible nitric oxide synthase
I/R: Ischemia/reperfusion
IRE1α: Inositol-requiring enzyme 1α
JNK: c-Jun N-terminal kinase
LIF: Leukemia inhibitory factor
Log P: Oil-in-water partition coefficient
LPHNPs: Lipid polymer hybrid nanoparticles
MAPKs: Mitogen-activated protein kinases
MCAO: Middle cerebral artery occlusion
NF-κB: Nuclear factor kappa-light-chain-enhancer of activated B cells
NGF: Nerve growth factor
NMCs: Natural medicines that affect cerebral metabolism
NMs: Natural medicines
Nrf-2: Nuclear factor erythroid 2-related factor 2
PPARα: Peroxisome proliferator-activated receptor-alpha
PPARγ: Peroxisome proliferator-activated receptor-gamma
sAPPα: Soluble amyloid precursor protein alpha
SULTs: Sulfotransferases
S100B: S100 calcium-binding protein B
TBI: Traumatic brain injury
TGF-β: Transforming growth factor-β
TLR4: Toll-like receptor 4
TPGS: D-α-tocopheryl polyethylene glycol 1000 succinate
TrkA: Tropomyosin receptor kinase A
TrkB: Tropomyosin receptor kinase B
UGTs: UDP-glucuronosyltransferases
VEGF: Vascular endothelial growth factor
