# Supplementary material for: Better Bioactivity, Cerebral Metabolism and Pharmacokinetics of Natural Medicine and Its Advanced Version
Source: Front Pharmacol. 2022 Jun 27;13:937075. doi: 10.3389/fphar.2022.937075 (PMC9271619; doi:10.3389/fphar.2022.937075)
Supplement: Supplementary file 1 [file DataSheet1.pdf]

Supporting Information

Better Bioactivity, Cerebral Metabolism and Pharmacokinetics of Natural Medicine and Its Advanced Version

Supplementary Table

Supplementary Table S1
Supplementary Table S2
Supplementary Table S3

Supplementary TABLE S1 | Pharmacological properties of NMCs.

Supplementary TABLE S2 | Physicochemical properties of NMCs.

Supplementary TABLE S3 | Metabolic reaction and metabolite of NMCs.

Supplementary TABLE S1 | Pharmacological properties of NMCs.

Structure classification	Drug	Indication	Neurotransmitter	Hormone	Neurotrophic molecule	Other brain-specific chemical
Terpene	Artemisinin	AD (Qiang et al., 2018)	Inhibit NO (Qiang et al., 2018)	-	Reduce IL-6 (Qiang et al., 2018)	Reduce NF-κB, IL-1β, TNF-α, TLR4 and MyD88 (Qiang et al., 2018)
Terpene	Tanshinone IIA	Focal cerebral ischemic injury (Zhu et al., 2017); Spastic cerebral palsy (Zhang et al., 2017); AD (He et al., 2020)	-	-	Active VEGF (Zhang et al., 2017)	Activate PI3K/Akt/mTOR pathway (Zhu et al., 2017); Suppress p-NF-κB, p-p38MAPK, inducible NOS and neuronal NOS, and activate the phosphorylation of inhibitor of NF-κB (Zhang et al., 2017); Reduce Aβ plaques and suppress the activation of CHOP and JNK pathways, prevent abnormal expression of GRP78, eIF2α, IRE1α, and ATF6 and upregulate the Bcl-2/Bax ratio and down-regulate caspase-3 (He et al., 2020)
Terpene	Ginsenoside Rb1	Cerebral ischemia (Guo et al., 2018); AD (Wang et al., 2018); Focal cerebral I/R injury (Zhao et al., 2018)	Reduce NO (Zhao et al., 2018)	-	Reduce IL-6 (Zhao et al., 2018)	Stimulate P-AKT/P-mTOR signaling pathway and inhibit P-PTEN protein (Guo et al., 2018); Decrease the levels of Bax and cleaved caspase-3 meanwhile increase the level of Bcl-2 in the hippocampus (Wang et al., 2018); Reduce TNF-α, NF-κB and inducible NOS, and inhibit HMGB1 (Zhao et al., 2018)
Terpene	Ginsenoside Rd (Yan et al., 2017)	AD (Yan et al., 2017)	-	-	-	Increase expression of α-secretase and sAPPα, while decrease β-secretase and Aβ (Yan et al., 2017)

Supplementary TABLE S1 | *(Continued)* Pharmacological properties of NMCs.

Structure classification	Drug	Indication	Neurotransmitter	Hormone	Neurotrophic molecule	Other brain-specific chemical
Terpene	Tetrandrine (Ren et al., 2021)	AD	-	-	Revers IL-6 (Ren et al., 2021)	Revers and inhibit IL-1β, TNF-α, iNOS, and NF-κB activity, and reduce Aβ plaques (Ren et al., 2021)
Terpene	Δ9-Tetrahydrocannabinol (Scott et al., 2014)	Glioma(Scott et al., 2014)	-	-	-	Active G protein–coupled cannabinoid receptors: CB1 and CB2 to stimulate MAPK and endoplasmic reticulum stress-related pathways(Scott et al., 2014)
Terpene	Cannabidiol (Watt and Karl, 2017)	AD(Watt and Karl, 2017)	Decrease NO (Watt and Karl, 2017)	-	Reduce IL-6 (Watt and Karl, 2017)	Inhibit S100B, iNOS, GFAP, tau hyperphosphorylation, NF-κB, IL-1β and disrupt the Wnt/β-catenin pathway(Watt and Karl, 2017)
Alkaloid	Capsaicin	AD(Xu et al., 2017)	-	-	-	Increase the activity of PI3K/AKT and inhibit GSK3β in hippocampus(Xu et al., 2017)
Alkaloid	Evodiamine	Cerebral ischemia (Zhao et al., 2014) ; Depression (Xu et al., 2021)	Increase the levels of noradrenaline and serotonin (Xu et al., 2021)	-	-	Upregulate pAkt and pGSK3β, and downregulate NF-κB expression(Tan and Zhang, 2016, Zhao et al., 2014)
Alkaloid	Leonurine	Depression (Jia et al., 2017); Cerebral ischemic stroke (Xie et al., 2019)	-	-	Inhibit IL-6 (Jia et al., 2017); Increase VEGF level (Xie et al., 2019)	Inhibit NF-κB, IL-1β, and TNF-α;(Jia et al., 2017)<br>Upregulate Nrf-2 protein expression(Xie et al., 2019)
Alkaloid	Oxymatrine	AD (Chen et al., 2019)	-	-	Reduce IL-6 (Chen et al., 2019)	Reduce IL-1β, TNF-α and IL-17A, and Aβ(Chen et al., 2019)
Alkaloid	Rhynchophylline	AD (Fu et al., 2021)	-	-	-	Blockade of EphA4 (Fu et al., 2021)

Supplementary TABLE S1 | *(Continued)* Pharmacological properties of NMCs.

Structure classification	Drug	Indication	Neurotransmitter	Hormone	Neurotrophic molecule	Other brain-specific chemical
Acid and ester	Salvianolic acid B	I/R-induced cerebral injury (Fan et al., 2018); AD (Tang et al., 2016); Depression (Zhang et al., 2016); Glioma (Wang et al., 2013)	-	-	Reduce IL-6 (Fan et al., 2018)	Reduce expression of GFAP, Iba1, IL-1β, TNF-α, and cleaved caspase-3(Fan et al., 2018); Decrease the protein expressions of BACE1 and sAPPβ and inhibit the activity of GSK3β(Tang et al., 2016); Decrease the expression of IL-1β and TNF-α while increase expression of IL-10 and TGF-β in the hippocampus and cortex(Zhang et al., 2016); Active p38-mediated ROS generation(Wang et al., 2013)
Acid and ester	Cholic acid	AD(Majid et al., 2019)	-	-	-	Stabilize the peptide of Aβ-42 and prevent its fibrillation(Majid et al., 2019)
Acid and ester	Butylphthalide	AD(Song et al., 2017)	-	-	-	Inhibit the expressions of MAPK(Song et al., 2017)
Vitamin A	Vitamin A	AD(Zeng et al., 2017)	-	-	-	Rescue Aβ production and neuritic plaque formation(Zeng et al., 2017)
Flavonoid	Rutin	Cerebral I/R injury(Liu et al., 2018)	-	-	Increase the levels of BDNF, NGF, TrkA, and TrkB in hippocampus and cerebral cortex(Liu et al., 2018)	Increase the level of ERα and ERβ(Liu et al., 2018)
Flavonoid	Scutellarein	AD(Huang et al., 2019)	-	-	-	Inhibit the AKT/ NF-κB signaling pathway(Huang et al., 2019)

Supplementary TABLE S1 | *(Continued)* Pharmacological properties of NMCs.

Structure classification	Drug	Indication	Neurotransmitter	Hormone	Neurotrophic molecule	Other brain-specific chemical
Flavonoid	Flavokawain B(Wang et al., 2018)	Glioma(Wang et al., 2018)	-	-	-	Protective autophagy through the ATF4-DDIT3-TRIB3-AKT-MTOR-RPS6KB1 signaling pathway(Wang et al., 2018)
Flavonoid (Glycoside)	Puerarin	AD(Yao et al., 2017)	-	-	-	Reduce tau hyperphosphorylation(Yao et al., 2017)
Flavonoid (Glycoside)	Icariin	AD, Cerebral ischemia, Depression(Jin et al., 2019)	Increase monoamine neurotransmitter levels and serotonin (depression)(Jin et al., 2019)	Decrease cortisol (depression) (Jin et al., 2019)	Reduce the protein level of TGF-β1 (cerebral ischemia)(Jin et al., 2019)	Prevent the production of $A\beta_{1-42}$ and inhibit APP and BACE-1 (AD), Suppress IκB-α degradation, NF-κB activation, and pro-inflammatory cytokine production, the and upregulate the expression of PPARα and PPARγ protein in brain tissues (cerebral ischemia), Decrease CRF, monoamine oxidase A and B activities, and serum CRF levels in brain tissues while reverse the decrease of GR and serotonin 1A receptor in the hippocampus and prefrontal cortex (depression) (Jin et al., 2019)
Glycoside	Baicalin	ADHD (Zhou et al., 2019); Depression (Guo et al., 2019); Depression (Lu et al., 2019)	Increase dopamine levels in the striatum(Zhou et al., 2019)	-	Decrease IL-6 (Guo et al., 2019); Increase BDNF and TrkB expression levels (Lu et al., 2019)	Increase the phosphorylation of PI3K, AKT and FoxO1 which inhibit the expression of TLR4 and decrease the levels of IL-1β, IL-6, and TNF-α in the hippocampus (Guo et al., 2019); Increase the level of synaptophysin and PSD95 (Lu et al., 2019)

Supplementary TABLE S1 | *(Continued)* Pharmacological properties of NMCs.

Structure classification	Drug	Indication	Neurotransmitter	Hormone	Neurotrophic molecule	Other brain-specific chemical
Glycoside	Geniposide	Depression (Zhao et al., 2018); AD(Liu et al., 2015)	Inhibit NO(Liu et al., 2015)	-	Reduce IL-6 (Liu et al., 2015)	Reduce the levels of IL-1β and TNF-α in the hippocampus, and restore the expression of GLP-1R/AKT signaling-related protein (Zhao et al., 2018); Reduce Aβ plaques and the release of TNF-α and IL-1β, inhibit phosphorylation of tau, suppress cyclooxygenase expression, and interfere expression of TLR4, while promote GLP-1R signaling pathway(Liu et al., 2015)
Glycoside	Oleandrin(Garofalo et al., 2017)	Glioma(Garofalo et al., 2017)	-	-	Enhance BDNF(Garofalo et al., 2017)	-
Phenylpropanoid	Curcumin	AD;(Tang and Taghibiglou, 2017) Depression (Fusar-Poli et al., 2020)	Increase of serotonin and dopamine(Fusar-Poli et al., 2020)	-	Increase BDNF(Fusar-Poli et al., 2020)	Inhibit formation and prompt disaggregation of Aβ and reduce hyperphosphorylation of tau and enhances its clearance (Tang and Taghibiglou, 2017); Inhibit monoamine oxidase A and B and NF-κB(Fusar-Poli et al., 2020)
Phenylpropanoid	Ferulic acid	Depression (Liu et al., 2017); Depression (Zheng et al., 2019); Depression (Sasaki et al., 2019)	Increase levels of dopamine and noradrenaline (Sasaki et al., 2019)	Decrease ACTH and corticosterone (Zheng et al., 2019)	Upregulate the levels of BDNF (Liu et al., 2017); Inhibit IL-6 (Zheng et al., 2019); Increase BDNF levels (Sasaki et al., 2019)	Upregulate the levels of PSD95 and synapsin I in the prefrontal cortex and hippocampus (Liu et al., 2017); Inhibit IL-1β and TNF-α, but increase the expression of IL-10 (Zheng et al., 2019)

Supplementary TABLE S1 | *(Continued)* Pharmacological properties of NMCs.

Structure classification	Drug	Indication	Neurotransmitter	Hormone	Neurotrophic molecule	Other brain-specific chemical
Phenylpropanoid	Chlorogenic acid	Focal cerebral I/R injury(Miao et al., 2017)	-	-	Increase NGF levels in brain tissue(Miao et al., 2017)	Increase EPO, HIF-1α(Miao et al., 2017)

Abbreviation: ACTH, adrenocorticotropin; AD, Alzheimer's disease; ADHD, attention deficit hyperactivity disorder; AKT, protein kinase b; APP, amyloid precursor protein; ATF4, activating transcription factor 4; ATF6, activating transcription factor 6; BACE1, beta-secretase 1; BDNF, brain-derived neurotrophic factor; CB1, cannabinoid receptor type 1; CB2, cannabinoid receptor type 2; CHOP, C/EBP homologous protein; CRF, corticotropin-releasing factor; DDIT3, DNA damage-inducible transcript 3; eIF2α, initiation factor 2α; EphA4, erythropoietin-producing hepatocellular A4; EPO, erythropoietin; ERα, estrogen receptor alpha; ERβ, estrogen receptor beta; FoxO1, forkhead box protein O1; GFAP, glial fibrillary acidic protein; GLP-1R, glucagon-like peptide-1 receptor; GR, glucocorticoid receptor; GRP78, glucose regulated protein 78; HIF-1α, hypoxia-inducible factor-α; HMGB1, high-mobility group box 1; I/R, ischemia/reperfusion; Iba1, ionized calcium-binding adaptor molecule 1; IL-10, interleukin 10; IL-17A, interleukin 17A; IL-1β, interleukin 1 beta; IL-6, interleukin 6; iNOS, inducible nitric oxide synthase; IRE1α, inositol-requiring enzyme 1α; IκB-α, nuclear factor of kappa light polypeptide gene enhancer in B-cells inhibitor, alpha; JNK, c-Jun N-terminal kinase; LPS, lipopolysaccharide; MAPK, mitogen-activated protein kinases; mTOR, mammalian target of rapamycin; MyD88, myeloid differentiation primary response 88; NF-κB, nuclear factor kappa-light chain-enhancer of activated B cells; NGF, nerve growth factor; NO, nitric oxide; NOS, nitric oxide synthase; Nrf-2, nuclear factor erythroid 2-related factor 2; p-GSK3β, phosphorylated glycogen synthase kinase 3 beta; p-TrkB, phosphorylated tropomyosin receptor kinase B; p38MAPK, p38 mitogen-activated protein kinases; PI3K, phosphoinositide 3-kinase; PPARα, peroxisome proliferator-activated receptors alpha; PPARγ, peroxisome proliferator-activated receptors gamma; PSD95, postsynaptic density protein 95; PTEN, phosphatase and tensin homolog; ROS, reactive oxygen species; RPS6KB1, ribosomal protein S6 Kinase b1; S100B, S100 calcium binding protein B; sAPPα, soluble amyloid precursor protein alpha; sAPPβ, soluble amyloid precursor protein beta; TGF-β, transforming growth factor beta; TLR4, toll-like receptor 4; TNF-α, tumor necrosis factor alpha; TRIB3, tribbles homolog 3; TrkA, tropomyosin receptor kinase A; TrkB, tropomyosin receptor kinase B; VEGF, vascular endothelial growth factor.

Supplementary TABLE S2 | Physicochemical properties of NMCs.

Drug	Structure classification	Structure and molecular weight	D_o	Log *P*	BCS
Artemisinin	Terpene	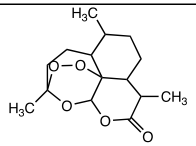 282.33 g/mol	-	3.53	II
Tanshinone IIA	Terpene	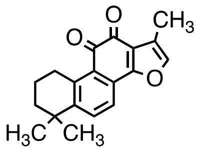 294.3 g/mol	57.14	4.93	II
Ginsenoside Rb$_1$	Terpene	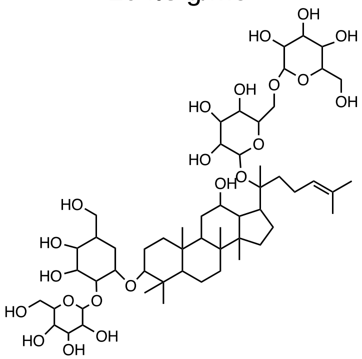 1109.3 g/mol	6792.45	1.88	II
Ginsenoside Rd	Terpene	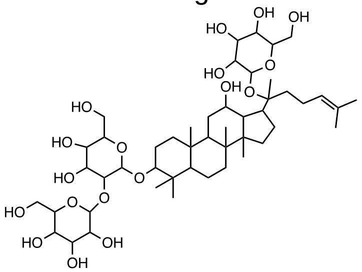 947.2 g/mol	1666.67	3.38	II

Supplementary TABLE S2 | *(Continued)* Physicochemical properties of NMCs.

Drug	Structure classification	Structure and molecular weight	D_o	Log *P*	BCS
Tetrandrine	Terpene	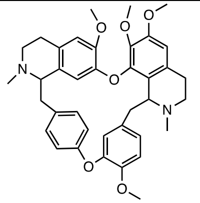 622.7 g/mol	-	5.55	II
Δ9-Tetrahydrocannabinol	Terpene	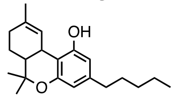 314.469 g/mol	-	5.648	II
Cannabidiol	Terpene	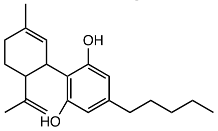 314.469 g/mol	-	6.1	II
Capsaicin	Alkaloid	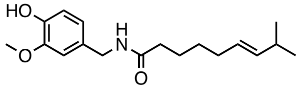 305.4 g/mol	12.00	4.00	II
Evodiamine	Alkaloid	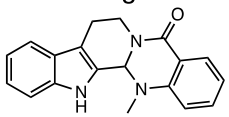 303.4 g/mol	-	-	II
Leonurine	Alkaloid	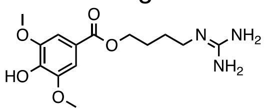 311.33 g/mol	5.71 E-02	7.20 E-01	III
Oxymatrine	Alkaloid	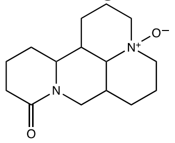 264.36 g/mol	-	9.50 E-01	III

Supplementary TABLE S2| *(Continued)* Physicochemical properties of NMCs.

Drug	Structure classification	Structure and molecular weight	D_o	Log *P*	BCS
Rhynchophylline	Alkaloid	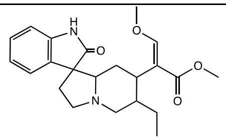 384.5 g/mol	-	-	-
Salvianolic acid B	Acid and ester	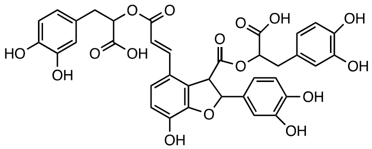 718.6 g/mol	2.95	2.14	II
Cholic acid	Acid and ester	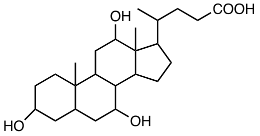 408.6 g/mol	4.62	2.88	II
Butylphthalide	Acid and ester	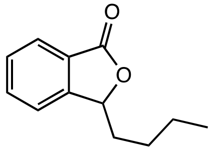 190.24 g/mol	6.15	3.05	II
Vitamin A	Vitamin	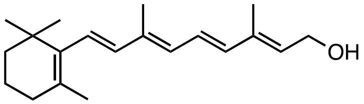 286.45 g/mol	44.00	6.40	II

Supplementary TABLE S2 | *(Continued)* Physicochemical properties of NMCs.

Drug	Structure classification	Structure and molecular weight	D_o	Log *P*	BCS
Rutin	Flavonoid	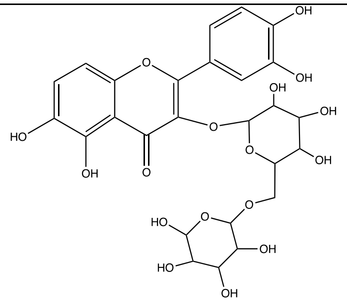 610.5 g/mol	2.87 E-02	-9.00 E-01	III
Scutellarein	Flavonoid	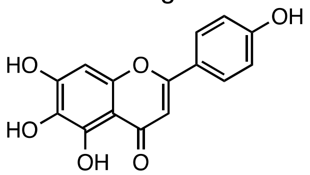 286.24 g/mol	2.00 E-04	4.30 E-01	III
Flavokawain B	Flavonoid	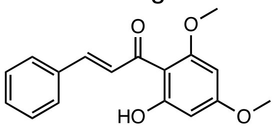 284.311 g/mol	-	-	-
Puerarin	Flavonoid (glycoside)	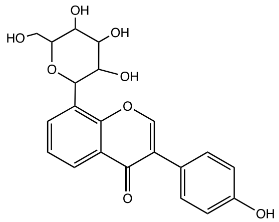 416.382 g/mol	1.33	0.48	VI

Supplementary TABLE S2 | *(Continued)* Physicochemical properties of NMCs.

Drug	Structure classification	Structure and molecular weight	D_o	Log *P*	BCS
Icariin	Flavonoid (glycoside)	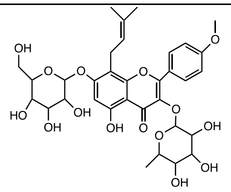 676.668 g/mol	13.33	0.71	VI
Baicalin	Glycoside	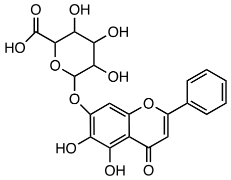 446.4 g/mol	8.00 E-04	1.43	III
Geniposide	Glycoside	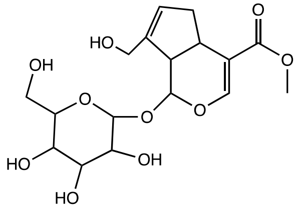 388.4 g/mol	1.00 E-04	-2.08	III
Oleandrin	Glycoside	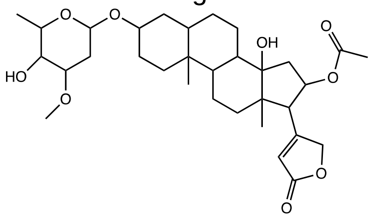 576.72 g/mol	-	2.1	-
Curcumin	Phenylpropanoid	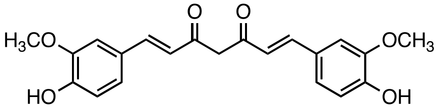 368.38 g/mol	-	3.62	II

Supplementary TABLE S2 | *(Continued)* Physicochemical properties of NMCs.

Drug	Structure classification	Structure and molecular weight	D_o	Log *P*	BCS
Ferulic acid	Phenylpropanoid	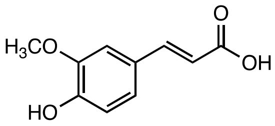 194.18 g/mol	1.00 E-04	9.60 E-01	III
Chlorogenic acid	Phenylpropanoid	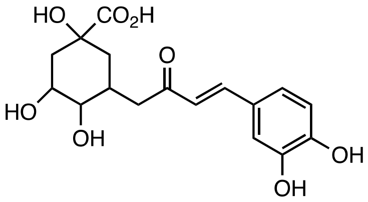 354.31 g/mol	1.00 E-04	3.70 E-01	III

Abbreviations: BCS, biopharmaceutical classification system; D_o, Dose number; *Log* P, oil in water partition coefficient

Note: - refers to not reported. The data of tetrandrine, tanshinone IIA, ginsenoside Rb1, ginsenoside Rd, capsaicin, salvianolic acid B, cholic acid, butylphthalide, vitamin A are collected based on the reference (Yan et al., 2018); the data of rutin, scutellarein, geniposide, leonurine, oxymatrine, ferulic acid, chlorogenic acid, and baicalin are collected based on the reference (Zeng et al., 2017); the data of puerarin and icariin are collected based on the reference (Yang et al., 2020); the data of oleandrin, curcumin, tetrandrine, Δ9-tetrahydrocannabinol, and cannabidiol are available on the website (https://go.drugbank.com/drugs).

Supplementary TABLE S3 | Metabolic reaction and metabolite of NMCs.

NMCs	Structure	Cerebral metabolites	Reaction type (Phase I or II)	Reference
Tanshinone IIA	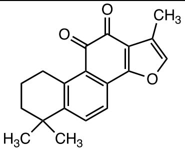	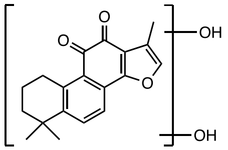 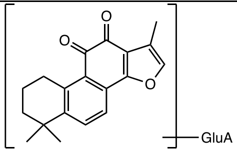	Double hydroxylation (Phase I)<br>Glucuronidation (Phase II)	(Liang et al., 2019)
Ginsenoside Rb1	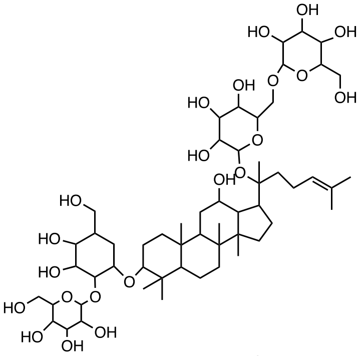	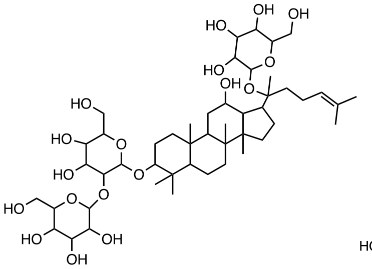 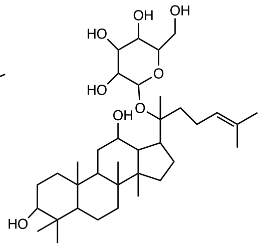	Deglycosylation (Phase I)	(Zhang et al., 2021)
Ginsenoside Rd	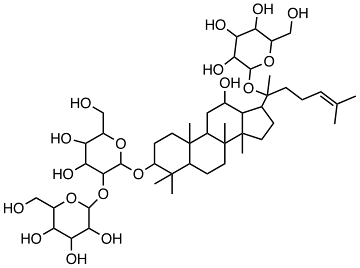	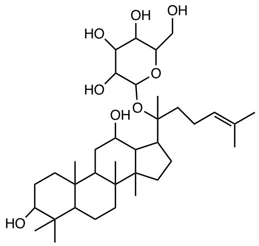	Deglycosylation (Phase I)	(Zhang et al., 2021)
Δ9-Tetrahydrocannabinol	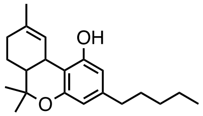	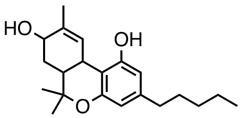 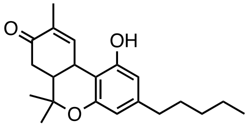 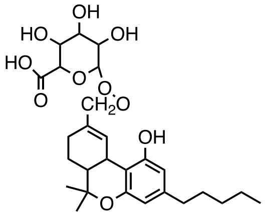	Hydroxylation (Phase I)<br>Oxidation (Phase I)<br>Glucuronidation (Phase II)	(Dinis-Oliveira, 2016)

Supplementary TABLE S3 | *(Continued)* Metabolic reaction and metabolite of NMCs.

NMCs	Structure	Cerebral metabolites	Reaction type (Phase I or II)	Reference
Capsaicin	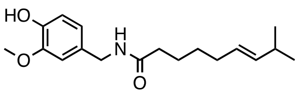	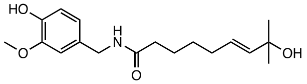 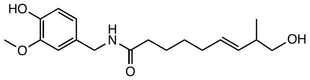 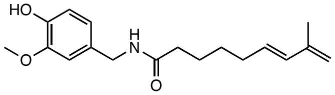 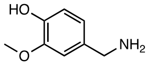	Hydroxylation (Phase I) Dehydrogenation (Phase I) Hydrolysis (Phase I)	(Rollyson et al., 2014)
Evodiamine	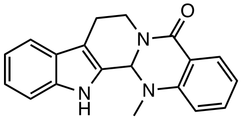	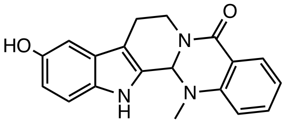 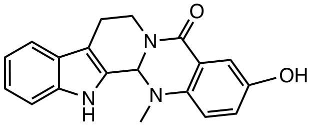 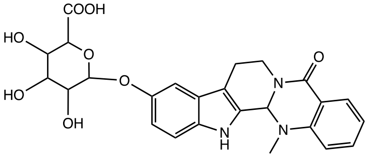 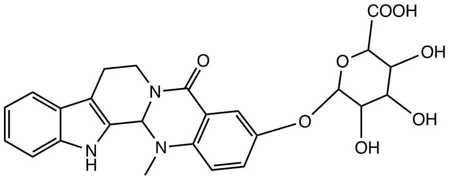	Hydroxylation (Phase I) Hydroxylation (Phase I) and glucuronide conjugation (Phase II)	(Wang et al., 2018)
Leonurine	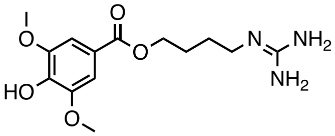	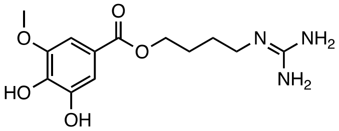 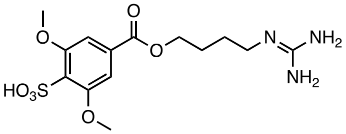 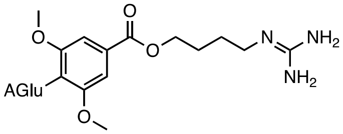	Demethylation (Phase I) Sulfonation (Phase II) Glucuronidation (Phase II)	(Zhu et al., 2014)

Supplementary TABLE S3 | *(Continued)* Metabolic reaction and metabolite of NMCs.

NMCs	Structure	Cerebral metabolites	Reaction type (Phase I or II)	Reference
Oxymatrine	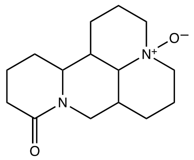	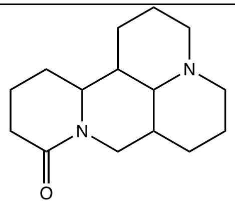	Reduction (Phase I)	(Liu et al., 2015)
Salvianolic acid B	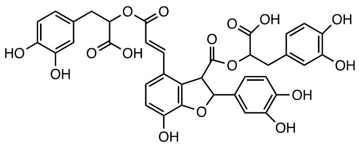	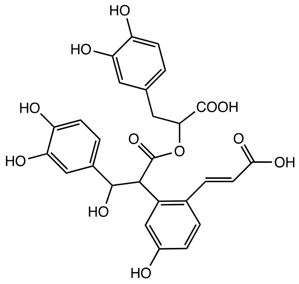 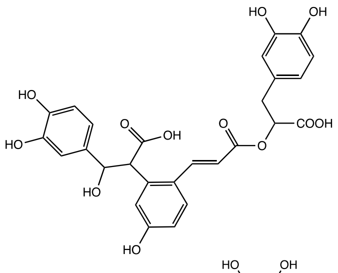 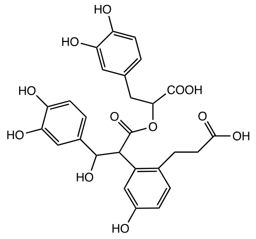 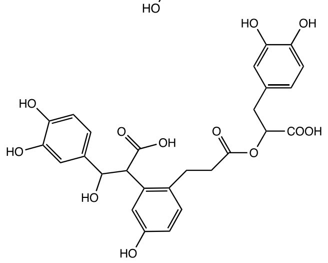	Hydrogenation (Phase I)	(Zhang et al., 2022)
		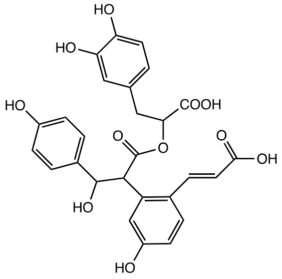 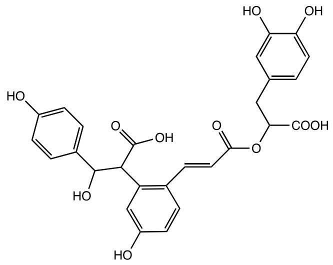	Hydrogenation + Dehydroxylation (Phase I)	

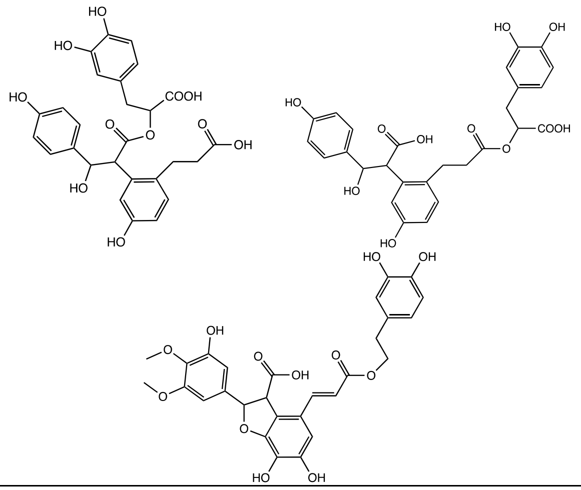

Methylation (Phase II) + Hydrogenation (Phase I) + Dehydroxylation (Phase I)

Supplementary TABLE S3 | *(Continued)* Metabolic reaction and metabolite of NMCs.

NMCs	Structure	Cerebral metabolites	Reaction type (Phase I or II)	Reference
Cholic acid	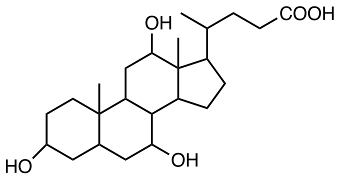	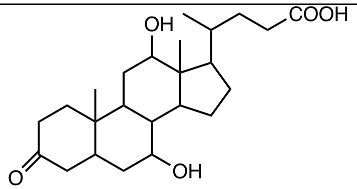	Dehydrogenation (Phase I)	(Funabashi et al., 2020)
Butylphthalide	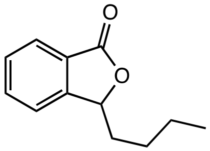	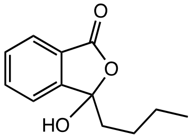 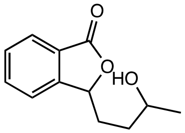	Hydroxylation (Phase I)	(Diao et al., 2015)
Vitamin A	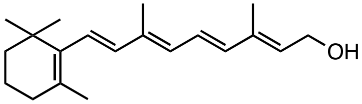	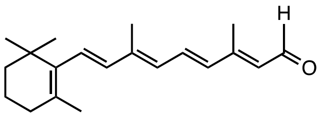 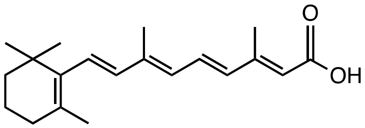	Dehydrogenation (Phase I)<br>Dehydrogenation + Hydroxylation	(Clugston and Blaner, 2014, Libien et al., 2017)
Rutin	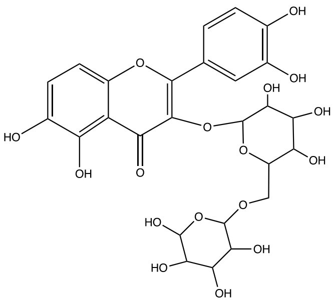	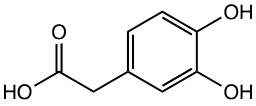 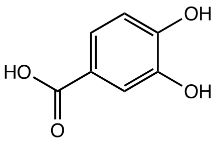 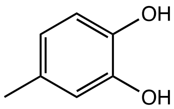 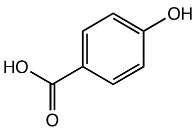 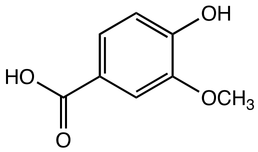	Hydrolysis (Phase I) Methylation (Phase II)	(Gimenez-Bastida et al., 2017, Morales et al., 2018)

Supplementary TABLE S3 | *(Continued)* Metabolic reaction and metabolite of NMCs.

NMCs	Structure	Cerebral metabolites	Reaction type (Phase I or II)	Reference
Scutellarein	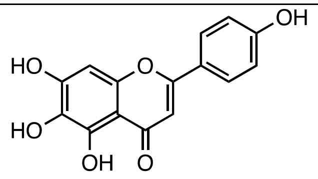	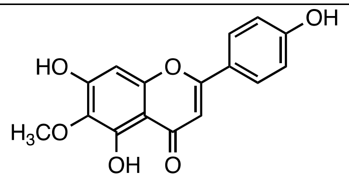	Methylation (Phase II)	(Shi et al., 2015)
Ferulic acid	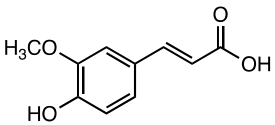	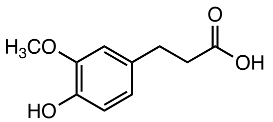 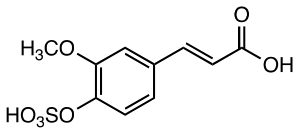 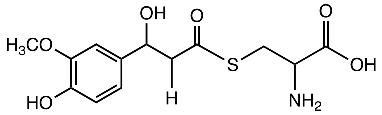 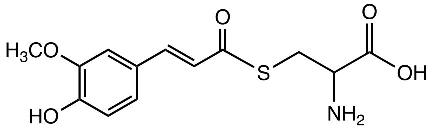 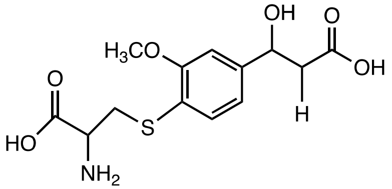	Hydrogenation (Phase I)<br>Sulphation (Phase II)<br>Cysteine conjugation (Phase II)	(Wang et al., 2016, Zhao et al., 2015)
Chlorogenic acid	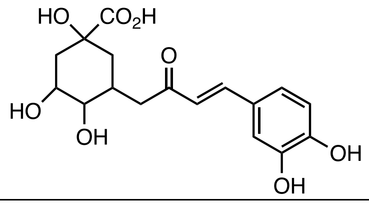	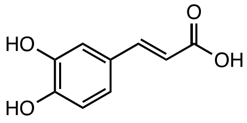 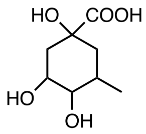	Hydrolysis (Phase I)	(Choi et al., 2018)

Supplementary TABLE S3 | *(Continued)* Metabolic reaction and metabolite of NMCs.

NMCs	Structure	Cerebral metabolites	Reaction type (Phase I or II)	Reference
Baicalin	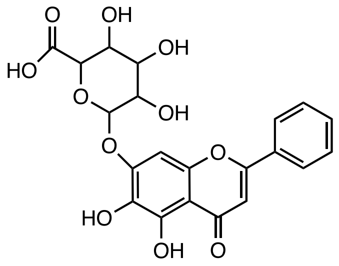	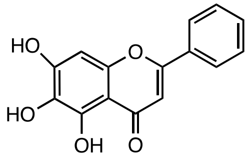 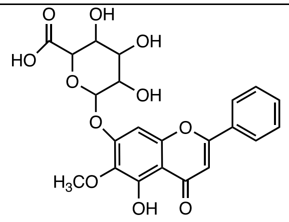 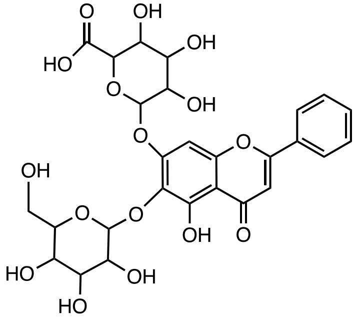	Deglycosylation (Phase I) Methylation (Phase II) Glucopyranoside conjugates (Phase II)	(Wang et al., 2017)
Geniposide	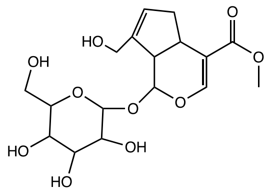	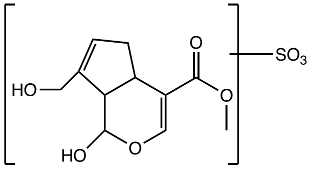 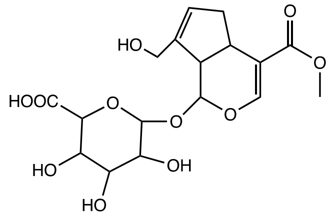	Hydrolysis (Phase I)+ sulfonation (Phase II) Hydrolysis (Phase I)+ glucuronidation (Phase II)	(Zhang et al., 2017)

Puerarin	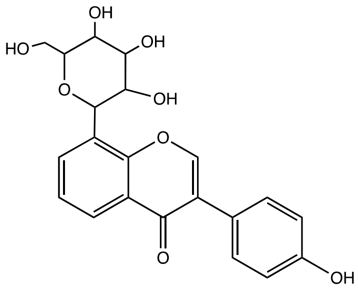	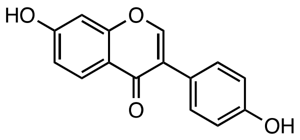 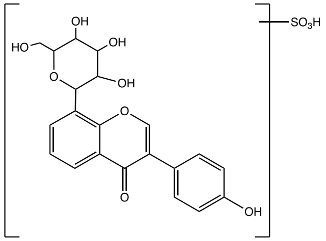	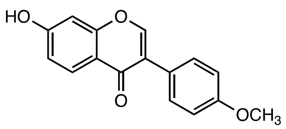 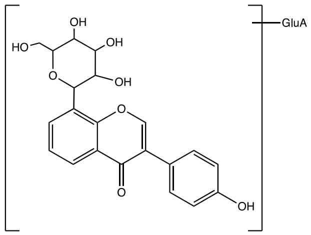	Deglycosylation (Phase I)<br>Methylation (Phase II)<br>Sulfonation (Phase II)<br>Glucuronidation (Phase II)	(Shang et al., 2017)

REFERENCES

Chen, Y., Qi, Z., Qiao, B., Lv, Z., Hao, Y., and Li, H. (2019). Oxymatrine Can Attenuate Pathological Deficits of Alzheimer's Disease Mice through Regulation of Neuroinflammation. *J Neuroimmunol* 334, 576978. doi: 10.1016/j.jneuroim.2019.576978

Choi, W.-G., Kim, J.-H., Kim, D., Lee, Y., Yoo, J., Shin, D., et al. (2018). Simultaneous Determination of Chlorogenic Acid Isomers and Metabolites in Rat Plasma Using Lc-Ms/Ms and Its Application to a Pharmacokinetic Study Following Oral Administration of Stauntonia Hexaphylla Leaf Extract (Yra-1909) to Rats. *Pharmaceutics* 10 (3). doi: 10.3390/pharmaceutics10030143

Clugston, R. D., and Blaner, W. S. (2014). Vitamin a (Retinoid) Metabolism and Actions: What We Know and What We Need to Know About Amphibians. *Zoo Biol* 33 (6), 527-535. doi: 10.1002/zoo.21140

Diao, X. X., Zhong, K., Li, X. L., Zhong, D. F., and Chen, X. Y. (2015). Isomer-Selective Distribution of 3-N-Butylphthalide (Nbp) Hydroxylated Metabolites, 3-Hydroxy-Nbp and 10-Hydroxy-Nbp, across the Rat Blood-Brain Barrier. *Acta Pharmacol Sin* 36 (12), 1520-1527. doi: 10.1038/aps.2015.64

Dinis-Oliveira, R. J. (2016). Metabolomics of Δ9-Tetrahydrocannabinol: Implications in Toxicity. *Drug Metabolism Reviews* 48 (1), 80-87. doi: 10.3109/03602532.2015.1137307

Fan, Y., Luo, Q., Wei, J., Lin, R., Lin, L., Li, Y., et al. (2018). Mechanism of Salvianolic Acid B Neuroprotection against Ischemia/Reperfusion Induced Cerebral Injury. *Brain Research* 1679, 125-133. doi: 10.1016/j.brainres.2017.11.027

Fu, W. Y., Hung, K. W., Lau, S. F., Butt, B., Yuen, V. W., Fu, G., et al. (2021). Rhynchophylline Administration Ameliorates Amyloid-Beta Pathology and Inflammation in an Alzheimer's Disease Transgenic Mouse Model. *ACS Chem Neurosci* 12 (22), 4249-4256. doi: 10.1021/acschemneuro.1c00600

Funabashi, M., Grove, T. L., Wang, M., Varma, Y., McFadden, M. E., Brown, L. C., et al. (2020). A Metabolic Pathway for Bile Acid Dehydroxylation by the Gut Microbiome. *Nature* 582 (7813), 566-570. doi: 10.1038/s41586-020-2396-4

Fusar-Poli, L., Vozza, L., Gabbiadini, A., Vanella, A., Concas, I., Tinacci, S., et al. (2020). Curcumin for Depression: A Meta-Analysis. *Crit Rev Food Sci Nutr* 60 (15), 2643-2653. doi: 10.1080/10408398.2019.1653260

Garofalo, S., Grimaldi, A., Chece, G., Porzia, A., Morrone, S., Mainiero, F., et al. (2017). The Glycoside Oleandrin Reduces Glioma Growth with Direct and Indirect Effects on Tumor Cells. *The Journal of Neuroscience* 37 (14), 3926-3939. doi: 10.1523/jneurosci.2296-16.2017

Gimenez-Bastida, J. A., Zielinski, H., Piskula, M., Zielinska, D., and Szawara-Nowak, D. (2017). Buckwheat Bioactive Compounds, Their Derived Phenolic Metabolites and Their Health Benefits. *Mol Nutr Food Res* 61 (7). doi: 10.1002/mnfr.201600475

Guo, L. T., Wang, S. Q., Su, J., Xu, L. X., Ji, Z. Y., Zhang, R. Y., et al. (2019). Baicalin Ameliorates Neuroinflammation-Induced Depressive-Like Behavior through Inhibition of Toll-Like Receptor 4 Expression Via the Pi3k/Akt/Foxo1 Pathway. *J Neuroinflammation* 16 (1), 95. doi: 10.1186/s12974-019-1474-8

Guo, Y., Wang, L. P., Li, C., Xiong, Y. X., Yan, Y. T., Zhao, L. Q., et al. (2018). Effects of Ginsenoside Rb1 on Expressions of Phosphorylation Akt/Phosphorylation Mtor/Phosphorylation Pten in

Artificial Abnormal Hippocampal Microenvironment in Rats. *Neurochem Res* 43 (10), 1927-1937. doi: 10.1007/s11064-018-2612-x

He, Y., Ruganzu, J. B., Lin, C., Ding, B., Zheng, Q., Wu, X., et al. (2020). Tanshinone Iia Ameliorates Cognitive Deficits by Inhibiting Endoplasmic Reticulum Stress-Induced Apoptosis in App/Ps1 Transgenic Mice. *Neurochem Int* 133, 104610. doi: 10.1016/j.neuint.2019.104610

Huang, X. W., Xu, Y., Sui, X., Lin, H., Xu, J. M., Han, D., et al. (2019). Scutellarein Suppresses Abeta-Induced Memory Impairment Via Inhibition of the Nf-Kappab Pathway in Vivo and in Vitro. *Oncol Lett* 17 (6), 5581-5589. doi: 10.3892/ol.2019.10274

Jia, M., Li, C., Zheng, Y., Ding, X., Chen, M., Ding, J., et al. (2017). Leonurine Exerts Antidepressant-Like Effects in the Chronic Mild Stress-Induced Depression Model in Mice by Inhibiting Neuroinflammation. *Int J Neuropsychopharmacol* 20 (11), 886-895. doi: 10.1093/ijnp/pyx062

Jin, J., Wang, H., Hua, X., Chen, D., Huang, C., and Chen, Z. (2019). An Outline for the Pharmacological Effect of Icariin in the Nervous System. *European Journal of Pharmacology* 842, 20-32. doi: 10.1016/j.ejphar.2018.10.006

Liang, S., Wang, Z., Yuan, J., Zhang, J., Dai, X., Qin, F., et al. (2019). Rapid Identification of Tanshinone Iia Metabolites in an Amyloid-Beta1-42 Induced Alzherimer's Disease Rat Model Using Uhplc-Q-Exactive Qrbitrap Mass Spectrometry. *Molecules* 24 (14). doi: 10.3390/molecules24142584

Libien, J., Kupersmith, M. J., Blaner, W., McDermott, M. P., Gao, S., Liu, Y., et al. (2017). Role of Vitamin a Metabolism in Iih: Results from the Idiopathic Intracranial Hypertension Treatment Trial. *J Neurol Sci* 372, 78-84. doi: 10.1016/j.jns.2016.11.014

Liu, H., Zhong, L., Zhang, Y., Liu, X., and Li, J. (2018). Rutin Attenuates Cerebral Ischemia-Reperfusion Injury in Ovariectomized Rats Via Estrogen-Receptor-Mediated Bdnf-Trkb and Ngf-Trka Signaling. *Biochem Cell Biol* 96 (5), 672-681. doi: 10.1139/bcb-2017-0209

Liu, W., Li, G., Holscher, C., and Li, L. (2015). Neuroprotective Effects of Geniposide on Alzheimer's Disease Pathology. *Rev Neurosci* 26 (4), 371-383. doi: 10.1515/revneuro-2015-0005

Liu, Y. M., Hu, C. Y., Shen, J. D., Wu, S. H., Li, Y. C., and Yi, L. T. (2017). Elevation of Synaptic Protein Is Associated with the Antidepressant-Like Effects of Ferulic Acid in a Chronic Model of Depression. *Physiol Behav* 169, 184-188. doi: 10.1016/j.physbeh.2016.12.003

Liu, Z., Liu, W., Shi, J., Zhu, L., Dong, L., Luo, F., et al. (2015). Reductive Metabolism of Oxymatrine Is Catalyzed by Microsomal Cyp3a4. *Drug Design, Development and Therapy*. doi: 10.2147/dddt.S92276

Lu, Y., Sun, G., Yang, F., Guan, Z., Zhang, Z., Zhao, J., et al. (2019). Baicalin Regulates Depression Behavior in Mice Exposed to Chronic Mild Stress Via the Rac/Limk/Cofilin Pathway. *Biomed Pharmacother* 116, 109054. doi: 10.1016/j.biopha.2019.109054

Majid, N., Siddiqi, M. K., Khan, A. N., Shabnam, S., Malik, S., Alam, A., et al. (2019). Biophysical Elucidation of Amyloid Fibrillation Inhibition and Prevention of Secondary Nucleation by Cholic Acid: An Unexplored Function of Cholic Acid. *ACS Chem Neurosci* 10 (11), 4704-4715. doi: 10.1021/acschemneuro.9b00482

Miao, M., Cao, L., Li, R., Fang, X., and Miao, Y. (2017). Protective Effect of Chlorogenic Acid on the Focal Cerebral Ischemia Reperfusion Rat Models. *Saudi Pharm J* 25 (4), 556-563. doi: 10.1016/j.jsps.2017.04.023

Morales, A. M., Mukai, R., Murota, K., and Terao, J. (2018). Inhibitory Effect of Catecholic Colonic Metabolites of Rutin on Fatty Acid Hydroperoxide and Hemoglobin Dependent Lipid Peroxidation in Caco-2 Cells. *J Clin Biochem Nutr* 63 (3), 175-180. doi: 10.3164/jcbn.18-38

Qiang, W., Cai, W., Yang, Q., Yang, L., Dai, Y., Zhao, Z., et al. (2018). Artemisinin B Improves Learning and Memory Impairment in Ad Dementia Mice by Suppressing Neuroinflammation. *Neuroscience* 395, 1-12. doi: 10.1016/j.neuroscience.2018.10.041

Ren, D., Fu, Y., Wang, L., Liu, J., Zhong, X., Yuan, J., et al. (2021). Tetrandrine Ameliorated Alzheimer's Disease through Suppressing Microglial Inflammatory Activation and Neurotoxicity in the 5xfad Mouse. *Phytomedicine* 90, 153627. doi: 10.1016/j.phymed.2021.153627

Rollyson, W. D., Stover, C. A., Brown, K. C., Perry, H. E., Stevenson, C. D., McNees, C. A., et al. (2014). Bioavailability of Capsaicin and Its Implications for Drug Delivery. *J Control Release* 196, 96-105. doi: 10.1016/j.jconrel.2014.09.027

Sasaki, K., Iwata, N., Ferdousi, F., and Isoda, H. (2019). Antidepressant-Like Effect of Ferulic Acid Via Promotion of Energy Metabolism Activity. *Mol Nutr Food Res* 63 (19), e1900327. doi: 10.1002/mnfr.201900327

Scott, K. A., Dalgleish, A. G., and Liu, W. M. (2014). The Combination of Cannabidiol and Delta9-Tetrahydrocannabinol Enhances the Anticancer Effects of Radiation in an Orthotopic Murine Glioma Model. *Mol Cancer Ther* 13 (12), 2955-2967. doi: 10.1158/1535-7163.MCT-14-0402

Shang, Z., Xin, Q., Zhao, W., Wang, Z., Li, Q., Zhang, J., et al. (2017). Rapid Profiling and Identification of Puerarin Metabolites in Rat Urine and Plasma after Oral Administration by Uhplc-Ltq-Orbitrap Mass Spectrometer. *J Chromatogr B Analyt Technol Biomed Life Sci* 1068-1069, 180-192. doi: 10.1016/j.jchromb.2017.10.038

Shi, Z. H., Li, N. G., Wang, Z. J., Tang, Y. P., Dong, Z. X., Zhang, W., et al. (2015). Synthesis and Biological Evaluation of Methylated Scutellarein Analogs Based on Metabolic Mechanism of Scutellarin in Vivo. *Eur J Med Chem* 106, 95-105. doi: 10.1016/j.ejmech.2015.10.039

Song, F. X., Wang, L., Liu, H., Wang, Y. L., and Zou, Y. (2017). Brain Cell Apoptosis Inhibition by Butylphthalide in Alzheimer's Disease Model in Rats. *Exp Ther Med* 13 (6), 2771-2774. doi: 10.3892/etm.2017.4322

Tan, Q., and Zhang, J. (2016). Evodiamine and Its Role in Chronic Diseases. *Adv Exp Med Biol* 929, 315-328. doi: 10.1007/978-3-319-41342-6_14

Tang, M., and Taghibiglou, C. (2017). The Mechanisms of Action of Curcumin in Alzheimer's Disease. *J Alzheimers Dis* 58 (4), 1003-1016. doi: 10.3233/JAD-170188

Tang, Y., Huang, D., Zhang, M. H., Zhang, W. S., Tang, Y. X., Shi, Z. X., et al. (2016). Salvianolic Acid B Inhibits Abeta Generation by Modulating Bace1 Activity in Sh-Sy5y-Appsw Cells. *Nutrients* 8 (6). doi: 10.3390/nu8060333

Wang, C., Yue, F., Ai, G., and Yang, J. (2018). Simultaneous Determination of Evodiamine and Its Four Metabolites in Rat Plasma by Lc-Ms/Ms and Its Application to a Pharmacokinetic Study. *Biomed Chromatogr* 32 (7), e4219. doi: 10.1002/bmc.4219

Wang, J., Qi, Q., Zhou, W., Feng, Z., Huang, B., Chen, A., et al. (2018). Inhibition of Glioma Growth by Flavokawain B Is Mediated through Endoplasmic Reticulum Stress Induced Autophagy. *Autophagy* 14 (11), 2007-2022. doi: 10.1080/15548627.2018.1501133

Wang, L., Huang, S., Chen, B., Zang, X. Y., Su, D., Liang, J., et al. (2016). Characterization of the Anticoagulative Constituents of Angelicae Sinensis Radix and Their Metabolites in Rats by Hplc-Dad-Esi-It-Tof-Msn. *Planta Med* 82 (4), 362-370. doi: 10.1055/s-0035-1558309

Wang, T., Jiang, H., Cao, S., Chen, Q., Cui, M., Wang, Z., et al. (2017). Baicalin and Its Metabolites Suppresses Gluconeogenesis through Activation of Ampk or Akt in Insulin Resistant Hepg-2 Cells. *Eur J Med Chem* 141, 92-100. doi: 10.1016/j.ejmech.2017.09.049

Wang, Y., Li, Y., Yang, W., Gao, S., Lin, J., Wang, T., et al. (2018). Ginsenoside Rb1 Inhibit Apoptosis in Rat Model of Alzheimer's Disease Induced by Aβ 1-40. *Am J Transl Res* 10 (3), 796-805.

Wang, Z. S., Luo, P., Dai, S. H., Liu, Z. B., Zheng, X. R., and Chen, T. (2013). Salvianolic Acid B Induces Apoptosis in Human Glioma U87 Cells through P38-Mediated Ros Generation. *Cell Mol Neurobiol* 33 (7), 921-928. doi: 10.1007/s10571-013-9958-z

Watt, G., and Karl, T. (2017). In Vivo Evidence for Therapeutic Properties of Cannabidiol (Cbd) for Alzheimer's Disease. *Front Pharmacol* 8, 20. doi: 10.3389/fphar.2017.00020

Xie, Y. Z., Zhang, X. J., Zhang, C., Yang, Y., He, J. N., and Chen, Y. X. (2019). Protective Effects of Leonurine against Ischemic Stroke in Mice by Activating Nuclear Factor Erythroid 2‐Related Factor 2 Pathway. *CNS Neuroscience & Therapeutics* 25 (9), 1006-1017. doi: 10.1111/cns.13146

Xu, D., Qiu, C., Wang, Y., Qiao, T., and Cui, Y. L. (2021). Intranasal Co-Delivery of Berberine and Evodiamine by Self-Assembled Thermosensitive in-Situ Hydrogels for Improving Depressive Disorder. *Int J Pharm* 603, 120667. doi: 10.1016/j.ijpharm.2021.120667

Xu, W., Liu, J., Ma, D., Yuan, G., Lu, Y., and Yang, Y. (2017). Capsaicin Reduces Alzheimer-Associated Tau Changes in the Hippocampus of Type 2 Diabetes Rats. *PLoS One* 12 (2), e0172477. doi: 10.1371/journal.pone.0172477

Yan, S., Liu, Y., Feng, J., Zhao, H., Yu, Z., Zhao, J., et al. (2018). Difference and Alteration in Pharmacokinetic and Metabolic Characteristics of Low-Solubility Natural Medicines. *Drug Metabolism Reviews* 50 (2), 140-160. doi: 10.1080/03602532.2018.1430823

Yan, X., Hu, G., Yan, W., Chen, T., Yang, F., Zhang, X., et al. (2017). Ginsenoside Rd Promotes Non-Amyloidogenic Pathway of Amyloid Precursor Protein Processing by Regulating Phosphorylation of Estrogen Receptor Alpha. *Life Sci* 168, 16-23. doi: 10.1016/j.lfs.2016.11.002

Yang, J., Li, K., He, D., Gu, J., Xu, J., Xie, J., et al. (2020). Toward a Better Understanding of Metabolic and Pharmacokinetic Characteristics of Low-Solubility, Low-Permeability Natural Medicines. *Drug Metabolism Reviews* 52 (1), 19-43. doi: 10.1080/03602532.2020.1714646

Yao, Y., Chen, X., Bao, Y., and Wu, Y. (2017). Puerarin Inhibits Β-Amyloid Peptide 1-42-Induced Tau Hyperphosphorylation Via the Wnt/Β-Catenin Signaling Pathway. *Molecular Medicine Reports* 16 (6), 9081-9085. doi: 10.3892/mmr.2017.7702

Zeng, J., Chen, L., Wang, Z., Chen, Q., Fan, Z., Jiang, H., et al. (2017). Marginal Vitamin a Deficiency Facilitates Alzheimer’s Pathogenesis. *Acta Neuropathologica* 133 (6), 967-982. doi: 10.1007/s00401-017-1669-y

Zeng, M., Yang, L., He, D., Li, Y., Shi, M., and Zhang, J. (2017). Metabolic Pathways and Pharmacokinetics of Natural Medicines with Low Permeability. *Drug Metabolism Reviews* 49 (4), 464-476. doi: 10.1080/03602532.2017.1377222

Zhang, F. X., Cui, S. S., Yuan, Y. L., Li, C., and Li, R. M. (2022). Dissection of the Potential Anti-Diabetes Mechanism of Salvianolic Acid B by Metabolite Profiling and Network Pharmacology. *Rapid Commun Mass Spectrom* 36 (1), e9205. doi: 10.1002/rcm.9205

Zhang, J. Q., Wu, X. H., Feng, Y., Xie, X. F., Fan, Y. H., Yan, S., et al. (2016). Salvianolic Acid B Ameliorates Depressive-Like Behaviors in Chronic Mild Stress-Treated Mice: Involvement of the Neuroinflammatory Pathway. *Acta Pharmacol Sin* 37 (9), 1141-1153. doi: 10.1038/aps.2016.63

Zhang, W., Cao, Y., Xia, J., Tian, L., Yang, L., and Peng, C. (2017). Neuroprotective Effect of Tanshinone Iia Weakens Spastic Cerebral Palsy through Inflammation, P38mapk and Vegf in Neonatal Rats. *Molecular Medicine Reports*. doi: 10.3892/mmr.2017.8069

Zhang, X., Chen, S., Duan, F., Liu, A., Li, S., Zhong, W., et al. (2021). Prebiotics Enhance the Biotransformation and Bioavailability of Ginsenosides in Rats by Modulating Gut Microbiota. *J Ginseng Res* 45 (2), 334-343. doi: 10.1016/j.jgr.2020.08.001

Zhang, X., Liu, S., Pi, Z., Liu, Z., and Song, F. (2017). Simultaneous Quantification Method for Comparative Pharmacokinetics Studies of Two Major Metabolites from Geniposide and Genipin by Online Mircrodialysis-Uplc-Ms/Ms. *J Chromatogr B Analyt Technol Biomed Life Sci* 1041-1042, 11-18. doi: 10.1016/j.jchromb.2016.12.010

Zhao, J., Liu, S., Hu, X., Zhang, Y., Yan, S., Zhao, H., et al. (2018). Improved Delivery of Natural Alkaloids into Lung Cancer through Woody Oil-Based Emulsive Nanosystems. *Drug Deliv* 25 (1), 1426-1437. doi: 10.1080/10717544.2018.1474970

Zhao, T., Zhang, X., Zhao, Y., Zhang, L., Bai, X., Zhang, J., et al. (2014). Pretreatment by Evodiamine Is Neuroprotective in Cerebral Ischemia: Up-Regulated Pakt, Pgsk3beta, Down-Regulated Nf-Kappab Expression, and Ameliorated Bbb Permeability. *Neurochem Res* 39 (8), 1612-1620. doi: 10.1007/s11064-014-1356-5

Zhao, X., Yang, D. H., Xu, F., Huang, S., Zhang, L., Liu, G. X., et al. (2015). The in Vivo Absorbed Constituents and Metabolites of Danshen Decoction in Rats Identified by Hplc with Electrospray Ionization Tandem Ion Trap and Time-of-Flight Mass Spectrometry. *Biomed Chromatogr* 29 (2), 285-304. doi: 10.1002/bmc.3275

Zhao, Y., Li, H., Fang, F., Qin, T., Xiao, W., Wang, Z., et al. (2018). Geniposide Improves Repeated Restraint Stress-Induced Depression-Like Behavior in Mice by Ameliorating Neuronal Apoptosis Via Regulating Glp-1r/Akt Signaling Pathway. *Neurosci Lett* 676, 19-26. doi: 10.1016/j.neulet.2018.04.010

Zheng, X., Cheng, Y., Chen, Y., Yue, Y., Li, Y., Xia, S., et al. (2019). Ferulic Acid Improves Depressive-Like Behavior in Prenatally-Stressed Offspring Rats Via Anti-Inflammatory Activity and Hpa Axis. *International Journal of Molecular Sciences* 20 (3). doi: 10.3390/ijms20030493

Zhou, R., Wang, J., Han, X., Ma, B., Yuan, H., and Song, Y. (2019). Baicalin Regulates the Dopamine System to Control the Core Symptoms of Adhd. *Molecular Brain* 12 (1). doi: 10.1186/s13041-019-0428-5

Zhu, Q., Zhang, J., Yang, P., Tan, B., Liu, X., Zheng, Y., et al. (2014). Characterization of Metabolites of Leonurine (Scm-198) in Rats after Oral Administration by Liquid Chromatography/Tandem Mass Spectrometry and Nmr Spectrometry. *ScientificWorldJournal* 2014, 947946. doi: 10.1155/2014/947946

Zhu, Y., Tang, Q., Wang, G., and Han, R. (2017). Tanshinone Iia Protects Hippocampal Neuronal Cells from Reactive Oxygen Species through Changes in Autophagy and Activation of

Phosphatidylinositol 3-Kinase, Protein Kinas B, and Mechanistic Target of Rapamycin Pathways. *Curr Neurovasc Res* 14 (2), 132-140. doi: 10.2174/1567202614666170306105315
